# Tailoring Phonon‐Driven Responses in α‐MoO_3_ through Isotopic Enrichment

**DOI:** 10.1002/adma.73629

**Published:** 2026-06-11

**Authors:** Thiago S. Arnaud, Ryan W. Spangler, Johnathan D. Georgaras, Jonah B. Haber, Daniel Hirt, Maximilian Obst, Gonzalo Álvarez‐Pérez, Mackey Long III, Felix G. Kaps, Jakob Wetzel, Courtney Ragle, John E. Buchner, Youngji Kim, Aditha S. Senarath, Richarda Niemann, Mingze He, Giulia Carini, Unai Arregui‐Leon, Akash C. Behera, Ramachandra Bangari, Nihar Sahoo, Niels C. Brumby, J. Michael Klopf, Martin Wolf, Lukas M. Eng, Susanne C. Kehr, Thomas G. Folland, Alexander Paarmann, Patrick E. Hopkins, Felipe Jornada, Jon‐Paul Maria, Joshua D. Caldwell

**Affiliations:** ^1^ Interdisciplinary Material Science Vanderbilt University Nashville Tennessee USA; ^2^ Department of Mechanical Engineering Vanderbilt University Nashville Tennessee USA; ^3^ Department of Materials Science and Engineering The Pennsylvania State University University Park Pennsylvania USA; ^4^ Department of Materials Science and Engineering Stanford University Stanford California USA; ^5^ Department of Mechanical and Aerospace Engineering University of Virginia Charlottesville Virginia USA; ^6^ Institute of Applied Physics TUD Dresden University of Technology Dresden Germany; ^7^ Würzburg‐Dresden Cluster of Excellence – EXC 2147 (ct.qmat) Dresden Germany; ^8^ Istituto Italiano di Tecnologia Center for Biomolecular Nanotechnologies Lecce Italy; ^9^ Department of Physcial Chemistry Fritz Haber Institute of the Max Planck Society Berlin Germany; ^10^ Department of Physics Politecnico di Milano Milan Italy; ^11^ Department of Physics and Astronomy University of Iowa Iowa City Iowa USA; ^12^ Institute of Radiation Physics Helmholtz‐Zentrum Dresden‐Rossendorf Dresden Germany; ^13^ Department of Materials Science and Engineering University of Virginia Charlottesville Virginia USA; ^14^ Department of Physics University of Virginia Charlottesville Virginia USA

**Keywords:** acoustic phonon, density functional perturbation theory, hyperbolic phonon polaritons, isotopic substitution, mid‐infrared, optical phonon, thermal conductivity

## Abstract

The implementation of polaritonic materials into nanoscale devices requires selective tuning of parameters to realize desired spectral or thermal responses. One robust material, α‐MoO_3_, an orthorhombic crystal boasting three distinct phonon dispersions, provides three polaritonic dispersions of hyperbolic phonon polaritons (HPhPs) across the mid‐infrared (MIR). Here, the tunability of both optical and thermal responses in isotopically enriched α‐MoO_3_ (^98^MoO_3,_ Mo^18^O_3_, and ^98^Mo^18^O_3_) is explored. A uniform ∼5% spectral redshift from ^18^O enrichment is observed in both Raman‐ and IR‐active TO phonons. Both the in‐ and out‐of‐plane thermal conductivities for the isotopic variations are reported. Ab initio calculations both replicate experimental findings and analyze the select‐mode three‐phonon scattering contributions. The HPhPs from each isotopic variation are probed with s‐SNOM, and we report an HPhP *Q*‐factor maxima increase in ^98^Mo^18^O_3_ of ∼50% along the [100] in the RB_2_ and ∼100% along the [001] in the RB_3_ with respect to ^98^MoO_3_. Observations in both real and Fourier space of higher‐order HPhP modes propagating in slabs of isotopically enriched α‐MoO_3_ without the use of a subdiffractional surface scatterer are presented here. This work establishes the dual‐element isotope enrichment of α‐MoO_3_ as an intrinsic strategy to design optical, thermal, and polaritonic properties.

## 1 Introduction

Polaritonic materials offer the subwavelength, directional, nanoscale confinement of light via polaritons—quasiparticles of light and matter that transport both energy and phase. Phonon polaritons are supported when a photon is coupled to the dipolar lattice oscillations (i.e., optical phonons) at a resonant frequency [1]. This resonant frequency is within the spectral regime known as the Reststrahlen band (RB), splitting between the transverse (TO) and longitudinal optical (LO) phonons, where the real parts of the dielectric permittivity tensor are negative.

Hyperbolic phonon polaritons (HPhPs) are a class of phonon polaritons with ray‐like propagation throughout the bulk of the crystal. As the name implies, HPhPs are supported within uniaxial (biaxial) hyperbolic crystals with spectral regions where at least one principal axes features a real part of the permittivity tensor that is negative, while at least one is positive [2]. HPhPs have been extensively studied in many uniaxial crystals, with the initial observations reported within hexagonal boron nitride [3, 4] (hBN), through launching mechanisms [5, 6], manipulation of substrate refractive index [7], hybrid modes within heterostructures [8, 9], modulation via highly doped semiconductor substrates [10], and hyperlensing [11, 12, 13, 14]. Molybdenum trioxide (α‐MoO_3_) is an orthorhombic van der Waals crystal with natural biaxial hyperbolicity that elicits anisotropic in‐plane HPhP propagation within the three Reststrahlen bands (RB_1‐3_) in the MIR and terahertz regime that each correspond to one of the three principal crystal directions [15, 16, 17]. Therefore, MoO_3_ intrinsically is capable of guiding in‐plane HPhPs between asymptotic limits along one crystallographic direction, while simultaneously restricting propagation in the others, leading to promise for applications in mid IR polarizers [18], polaritonic‐driven chemistry [19], in‐plane HPhP manipulation [20, 21, 22, 23, 24], nanoresonators based on negative reflection [25], negative refraction [26], geometric confinement [27], substrate mediation [28, 29], twist‐optics [9, 30, 31, 32, 33, 34, 35, 36], chiral emission [37], hypercrystals [38], and gate tuning HPhPs coupled with graphene plasmons [39, 40, 41].

Aside from tuning HPhPs through the external environment [7], isotopic enrichments offer an avenue to intrinsically control polariton lifetimes and dispersion. *Caldwell* et al. discussed the significance of isotope enrichment in a diatomic crystal, where the acoustic phonon lifetimes are primarily dictated by the heavier mass and the optic phonon lifetimes are mostly dependent on the lighter mass [1]. Eliminating the isotopic disorder present in naturally abundant crystals inherently lowers the HPhP loss through reduced scattering rates. *Giles* et al. demonstrated a 3–4 fold improvement in the optic phonon lifetime in isotopically enriched hBN, which translated into corresponding improved HPhP lifetimes and propagation lengths [42]. While the optic phonon lifetime improvements were shown through the low‐loss HPhPs supported within isotopically enriched hBN, the small differences in atomic mass between boron and nitrogen makes it difficult to discern the atomic dependence between optic and acoustic modes. A recent study performed by *Carini* et al. observed a ∼40 cm^−1^ redshift in phonon frequencies in monoclinic β‐Ga_2_O_3_ (bGO) when isotopically substituting ^16^O with ^18^O [43]. Similar to bGO, α‐MoO_3_ also has a large atomic mass difference, which serves as an excellent platform to unveil specific spectral responses and modifications in the thermal response via selective isotopic enrichment.

Naturally abundant MoO_3_ (nat‐MoO_3_) is comprised of seven stable Mo isotopes with significant concentrations: ^92^Mo (15.86%), ^94^Mo (9.12%), ^95^Mo (15.70%), ^96^Mo (16.50%), ^97^Mo (9.45%), ^98^Mo (23.75%), and ^100^Mo (9.62%) [44]. Zhao et al. studied HPhPs within RB_3_ supported along the [100] in isotopically pure ^92^MoO_3_ and ^100^MoO_3_ and reported improved propagation lengths and lifetimes for both isotopes in comparison to naturally abundant MoO_3_ [45]. Schultz et al. later performed a similar investigation of the same Mo isotopes within RB_2_, realizing modest improvements in propagation lengths and lifetimes [46]. However, prior works have not explored the implementation of oxygen isotopic enrichment to modify HPhP responses. This is a critical distinction, as the optic phonon frequencies and damping, and therefore, the HPhP properties are most sensitive to the lighter atomic mass [1]. Moreover, switching between ^16^O and ^18^O isotopic variations in α‐MoO_3_ would better reveal the role of the Mo on both optically and thermally driven properties. More importantly so, a comprehensive study has yet to experimentally investigate the individual atomic mass dependences in α‐MoO_3_ and differentiate their respective influences upon the acoustic and optic phonon‐driven responses.

Here we reveal the anisotropic losses present in the MIR optical phonons of α‐MoO_3_ via isotopic enrichment of both oxygen and molybdenum with respect to natural abundant α‐MoO_3_. We prepare samples of varying isotopic content: ^98^MoO_3,_ Mo^18^O_3_ and ^98^Mo^18^O_3_ to investigate implications of a ∼12.5% mass increase in the lighter mass and further study the role of the reduced Mo isotopic disorder plays in the optical and thermal properties. In the far‐field regime, we observe significant phonon frequency red shifts due to ^18^O enrichment from Raman and Fourier transform infrared (FTIR) spectroscopy. Regarding the thermal properties, we utilize time‐domain thermoreflectance (TDTR) to extract the thermal conductivity from each isotopic sample along the in‐ and through‐plane directions. We find good agreement between first‐principles density functional perturbation theory (DFPT) calculations and the thermal and far‐field optical experimental results. We provide additional DFT analysis on the acoustic and optical phonon scattering processes governed by their respective scattering phase‐space and the corresponding mode‐dependent coupling strength (set by their eigenvectors and degree of Mo─O atomic character). Shifting to the near‐field regime, we probe HPhPs in all three MIR RBs to fully characterize the polaritonic nature of isotopically enriched α‐MoO_3_. Notable findings include relative increases in HPhP *Q*‐factor with respect to the phonon damping in the RB_2_ and RB_3_. Additionally, we found that, within the RB_3_, the in‐plane direction that supports higher *Q* HPhPs flip when isotopically enriched. Furthermore, we report the direct, real‐space imaging of higher order modes in single slabs of isotopic α‐MoO_3_. This work investigates the interplay between Mo and O isotopic enrichment in α‐MoO_3_, which enables both the spectral tuning of phonon frequencies and the in‐plane anisotropic enhancement of HPhP *Q*‐factors to enhance its applications in MIR polarizers, chiral emission, and nanoscale confinement and manipulation of both light and heat.

## 2 Results

**FIGURE 1:**
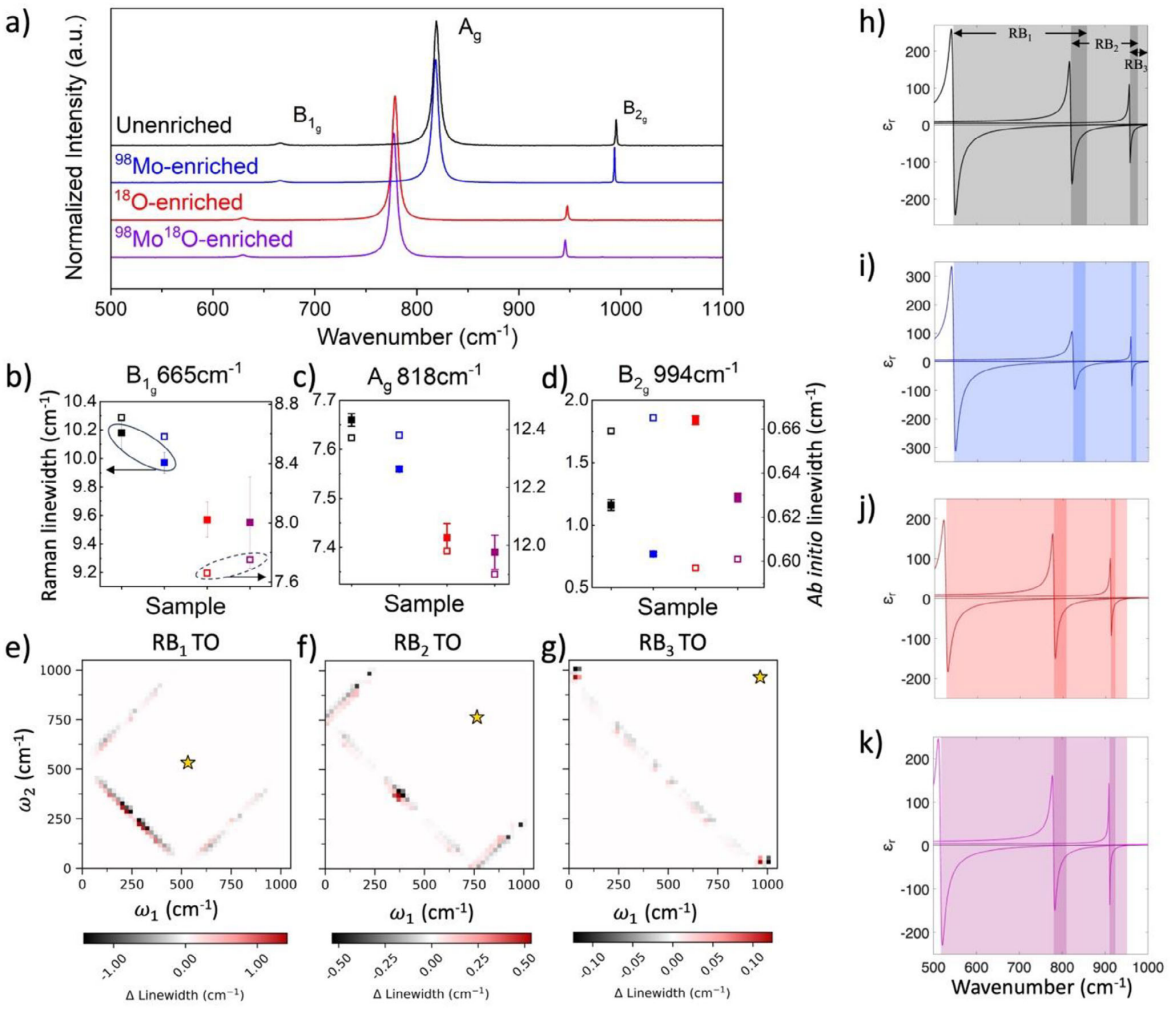
Far‐field optical characterization on varying isotopic contents of α‐MoO_3_. (a) Raman spectroscopy for each labeled isotopically enriched sample. The Raman modes are labeled beside their respective peaks. The same isotope‐color scheme in part (a) is repeated throughout the rest of Figure 1. (b–d) Extracted MIR Raman linewidths from the modes labeled in (a) and their frequencies with respect to naturally abundant (unenriched) α‐MoO_3_. The horizontal axis is indexed by sample for clarity since the peaks would be tightly grouped in frequency for crystals of ^16^O and ^18^O content. The error bars are ±1 standard deviation. (e–g) Mode‐resolved *difference histograms* showing the enrichment‐induced redistribution of the three‐phonon contributions to the intrinsic linewidth upon ^18^O substitution for the corresponding Γ‐point modes (RB_1‐3_ TO phonons marked by the gold star). The axes represent the frequencies of the daughter phonon pairs (*ω*_1_, *ω*_2_) involved in the three‐phonon decay and coalescence processes. Each (*ω*_1_, *ω*_2_) bin aggregates the summed three‐phonon linewidth contributions from all momentum/branch contributions whose daughter frequencies fall within that bin. The color scale indicates the change of the linewidth contributions in cm^−1^: black (negative) regions correspond to reduced contributions under ^18^O enrichment, while red (positive) regions indicate increased contributions. (h–k) Experimentally approximated real‐part of the dielectric functions for each isotopic variation of α‐MoO_3_. The parameters for the dielectric function of naturally abundant α‐MoO_3_ are taken from reported values [15].

Reactive vapor transport was used to grow α‐MoO_3_ crystals of varying isotopic contents (see methods): naturally abundant α‐MoO_3_, ^98^MoO_3_, Mo^18^O_3_, and ^98^Mo^18^O_3_. The ^98^Mo isotope was selected out of the seven stable options due to it being the largest quantity present in natural abundant α‐MoO_3_ with the intent of establishing an enriched sample closest to naturally abundant MoO_3_ without the isotopic disorder scattering loss or additional spectral shifts contributed from other Mo isotopes. The near monoisotopic crystals were then characterized using Raman spectroscopy (see methods), where a systematic ∼ 5% red shift from naturally abundant MoO_3_ was observed in both Mo^18^O_3_ and ^98^Mo^18^O_3_ (Figure 1a). It is important to note that the percent red shift from oxygen enrichment does not hold for acoustic phonons below ∼190 cm ^−1^ as seen in the DFPT calculated phonon dispersion (Figure S1.1; see methods). These shifts in the higher energy phonons are in agreement with reported Raman shifts from ^18^O enriched bGO [47]. Beyond the simple difference in relative mass increase (∼ 12.5% for O vs ∼ 2% for Mo), we identify that these MIR optic phonons are predominantly oxygen in character (see Figure S1.2), making them exceptionally sensitive to detuning via oxygen substitution. Comparison between the measured and Ab initio linewidths of the TO Raman peaks reveal excellent agreement between expected and observed linewidth reductions from ^18^O enrichment (Figure 1b–d). The full dependence of these mechanisms on optical mode character and atomic participation is further discussed in Section S2. We note that the apparent discrepancies between the experimental and theoretical values in Figure 1d—particularly for the narrow B_2g_ mode—is a result of the experimental spectral resolution limit of 0.8 cm^−1^ (see methods). Since the predicted intrinsic anharmonic linewidth of the B_2g_ mode (≈0.66 cm^−1^) falls below this instrumental threshold, the experimental measurements are instrument‐limited and cannot fully resolve the fine linewidth narrowing predicted by theory. However, the theoretical trends remain consistent with the physical picture of moderate lifetime improvements from ^18^O enrichment.

To correlate our far‐field Raman measurements and make appropriate near‐field approximations, we characterize the IR‐active phonons through FTIR spectroscopy (see methods and Section S3 for more details). Reflection spectra were collected at near‐normal incidence polarized along the [100] and [001] directions, showing the characteristic high reflectivity Reststrahlen bands of the RB_1_ and RB_2_ plotted in Figure S3.2a, respectively. Due to the angular spread present in the reflective optics in the measurements, we observe additional dips in the reflectivity that correspond to the out‐of‐plane RB_3_ TO phonon and additional modes further discussed from Figure S3.2b. Additional measurements were taken at an oblique angle of incidence to increase sensitivity to the out‐of‐plane RB_3_. (Figure S3.2c,d) Similar to the Raman spectra, redshifts of ∼ 5% in the IR‐active TO phonons are observed due to isotopic enrichment of ^18^O in α‐MoO_3_. As previously mentioned, near equivalent shifts in the RBs were observed in ^18^O enriched bGO [43]. Such agreement in ^18^O studies in different crystals clearly supports the idea that the optic phonons are strongly governed by the lighter mass in a diatomic crystal.

Rather than identifying the scattering channels contributing to each TO phonon linewidth, as done with the Raman modes in Section S2, we focus on the *change* in three‐phonon scattering contributions between MoO_3_ and Mo^18^O_3_. Thus, we investigate the microscopic origin of the linewidth narrowing under ^18^O enrichment via the linewidth difference histograms for the RB_1‐3_ TO phonons (parent‐phonon frequencies) shown in Figure 1e–g. The difference maps report the change in the mode‐resolved three‐phonon contribution to the intrinsic linewidth when isotopically substituting from ^16^O to ^18^O. The axes represent the daughter‐phonon frequency pairs (*ω*_1_, *ω*_2_) that participate in energy‐ and momentum‐conserving decay and coalescence processes. Consistent with the measured narrowed linewidths, all three modes exhibit a net reduction in intrinsic linewidth under ^18^O enrichment (RB_1‐3_ TO: ΔΓ_1‐3_ = −2.66, −1.16, and −0.114 cm^−1^, corresponding to −11.8%, −15.0%, and −11.5%, respectively), accompanied by substantial redshifts of the parent frequencies (Δω_0_ ≈ − 25 to − 42 cm^−1^).

The phonon‐phonon scattering rate inherits a $1/\sqrt{m}$ mass factors from eigenvector normalization, so heavier isotope substitution might naively be expected to uniformly reduce all anharmonic decay rates [48]. However, the difference histograms contain both positive and negative regions, indicating that ^18^O substitution redistributes the three‐phonon scattering rather than uniformly suppressing it or simply redshifting the available energy‐conserving phase‐space. The net linewidth reduction from ^16^O to ^18^O arises from an incomplete cancellation between the suppressed channels (negative regions) and enhanced channels (positive regions), with the former outweighing the latter. The mode‐dependent redistribution is seen in the difference between linewidth changes of the RB_1‐3_ TO phonons: the linewidth decrease of the RB_1_ TO phonon is dominated by reduced contributions involving intermediate daughter frequencies (ω ≤ 500 cm^−1^); the RB_2_ TO phonon shows pronounced suppression for mixed low‐ and high‐frequency daughter pairs; and the RB_3_ TO phonon exhibits a characteristic transfer of scattering weight away from the upper edge of the phonon spectrum (ω ≥ 1000 cm^−1^) toward slightly lower daughter frequencies. These mode‐dependent trends arise from non‐uniform softening of the phonon spectrum, where the frequencies of different branches shift by different amounts under ^1^ ^8^O enrichment, reshaping the energy‐conserving phase‐space and redistributing the dominant three‐phonon pathways.

The IR optical characteristics of α‐MoO_3_ are captured in its dielectric function and, in the absence of free carriers, arise mainly from the various IR phonon resonances. The dielectric function for TO and LO splittings is described by a Lorentz oscillator for each principal axis in the diagonal permittivity tensor [15]:

(1) $$\varepsilon_{j}\left(\omega \right)=\varepsilon_{j}^{\infty }\left(\frac{{\left(\omega_{j}^{LO}\right)}^{2}-\omega^{2}-i\gamma_{j}\omega }{{\left(\omega_{j}^{TO}\right)}^{2}-\omega^{2}-i\gamma_{j}\omega }\right),\;j=x,y,z$$

where ε_*j*_(ω) is the permittivity component denoted by *j* along the [100], [001], and [010] for x, y, and z, respectively, $\varepsilon_{j}^{\infty }$ is the high‐frequency dielectric constant, $\omega_{j}^{TO}$ and $\omega_{j}^{LO}$ are the TO and LO phonon frequencies, and γ_*j*_ is the phonon damping.

Since this study investigates isotopically enriched α‐MoO_3_ not previously reported, we provide their respective optical behaviors here. As a first approximation of the dielectric function for each isotopic sample, we alter the TO and LO phonons from naturally abundant α‐MoO_3_ according to their respective Raman and IR spectral shifts observed. Knowing the TO and LO frequencies from experimental data and approximate shifts, we utilize the Lyddane‐Sachs‐Teller relation [49]:

(2) $$\frac{\varepsilon_{0}}{\varepsilon_{j}^{\infty }}=\left(\frac{{\left(\omega_{j}^{LO}\right)}^{2}}{{\left(\omega_{j}^{TO}\right)}^{2}}\right),\;j=x,y,z$$

to estimate the $\varepsilon_{j}^{\infty }$ value for each direction with the known TO and LO frequencies and the dielectric constant, ε_0_. The phonon damping for each isotopically enriched sample is fixed with reported values of naturally abundant α‐MoO_3_ values [15] due to a recent study from Nandanwar et al. [50], which revealed overestimates in phonon damping from isotopic hBN values reported by Giles et al. [42] for measurements at room temperature. In consideration of all these factors, we insert the redshifted TO and LO parameters into Equation (1) and plot the approximated dielectric functions for each isotopically enriched α‐MoO_3_ in Figure 1h–k. While γ is kept at the naturally abundant α‐MoO_3_ value and therefore, not plotted, we can infer from the Raman linewidth reductions and DFPT difference scattering phase‐space that the IR‐active TO linewidths also experience similar reductions from ^18^O enrichment. These conclusions are further supported by DFPT calculated TO linewidths (Figure S4.1). Therefore, we expect modest phonon lifetime improvements in the ^18^O flakes but, it is difficult to discern between phonon‐phonon scattering and additional scattering losses arising from isotopic disorder.

With strong experimental evidence indicating the far‐field optic phonon characteristics dependent on the lighter of the two masses, we also examine the influence of isotopic enrichment on the lower frequency, heat carrying optic and acoustic phonon properties contingent upon the heavier mass. To this extent, we perform time‐domain thermoreflectance (TDTR; see Section S5) measurements to extract the in‐ and out‐of‐plane thermal conductivity from each of our isotopically enriched α‐MoO_3_ flakes. The out‐of‐plane (in‐plane) thermal conductivities on each sample are listed in Table S5.1 (S5.4) alongside the DFPT calculated values (see Section S1.4 for more details). Further information is available in Section S5 regarding necessary fittings, uncertainty analysis and thermal boundary conductance. Our measured thermal conductivities reflect the predicted thermal conductivity behavior for a vdW crystal, exhibiting higher thermal conductivity in the in‐ vs out‐of‐plane directions. We observe similar thermal conductivities across our available isotopes in this study due to little variance between the Mo atomic weight present in naturally abundant α‐MoO_3_ (∼95.96 u) and the 98 u enriched flake. This equates to a ∼2% change in Mo mass compared to the ∼12% change in O mass from ^16^O to ^18^O. This is consistent with prior works [51], given the relative reduction in phonon scattering rates for lower frequency phonons to changes in atomic mass as compared to the higher frequency, non‐thermally activated optically active phonons [52].

We further investigate the thermal conductivity of α‐MoO_3_ through Ab initio calculations. The Ab initio thermal conductivity values plotted in Figure S6.1 are in great agreement with the experimental values reported in Table S5.1 and S5.4. We employ the same eigenvector atomic projections onto the phonon dispersion to identify the acoustic and optic phonon contributions driven by Mo or O atoms (Figure S6.2). We correlate both projections upon the phonon dispersion to identify anisotropic pathways of thermal transport that can be isotopically tuned to the degree of Mo or O character. As a result, we predict a significant ∼ 30% contribution from the optic phonons and a ∼ 40% contribution specifically from the oxygen atoms in both the in‐ and cross‐plane thermal transport. We breakdown these theoretical thermal conductivity predictions into their independent contributions from phonon heat capacity, phonon group velocity, phonon‐phonon scattering rates, and isotope scattering (Figure S6.3). The significant role of optic phonons upon the thermal conductivity implies that the isotopic enrichment of the lighter mass counterintuitively plays an important role. Through the strong control of optic phonon dispersion from ^18^O enrichment, we directly influence the lifetimes by reshaping the three‐phonon scattering phase space, as previously discussed in Figure 1e–g, and indirectly influence the thermal transport. Thus, this platform of dual‐element enriched MoO_3_ offers a new tuning knob that was not previously explored to tune both the thermal and optical properties by the manipulation of optic phonon lifetimes. Recent work has explored the ultrafast thermal transport across the Au/hBN interface mediated by the excitation of HPhPs in hBN [53]. Similar future work should explore the role of the in‐plane anisotropy intrinsic to α‐MoO_3_ upon directional, nanoscale thermal transport mediated by isotopic HPhPs that is beyond the scope of this work.

**FIGURE 2:**
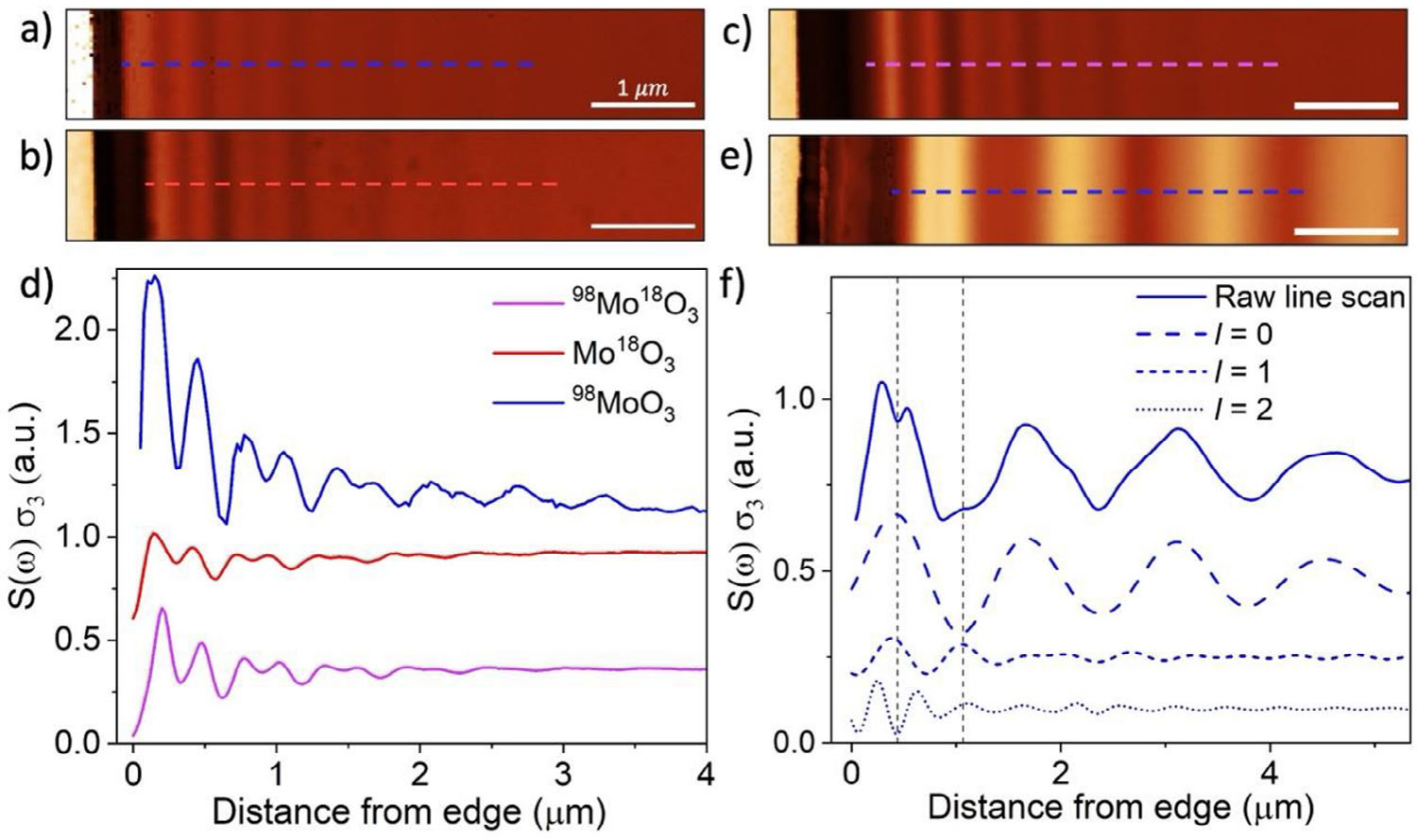
Experimental near‐field characterization of varying isotopic content of α‐MoO_3_. (a–c) Experimental SNOM measurements of similar thickness (∼200 nm) ^98^MoO_3_ at 920 cm^−1^, Mo^18^O_3_ at 880 cm^−1^, and ^98^Mo^18^O_3_ at 880 cm^−1^, respectively. The dashed lines illustrate where the HPhP line profiles are taken from the SNOM images; correlated with AFM profiles at the start of the surface from the flake edge. (d) Extracted line profiles from (a–c) for HPhPs of similar wavelength. (e) SNOM measurement of a 460 nm ^98^MoO_3_ flake at 880 cm^−1^ where higher‐order modes are visibly present in the real‐space image. (f) Deconvolved FFT‐filtered line profile of (e), where the solid line is the raw extracted profile, and subsequent profiles below are increasing in higher‐order HPhP. The vertical lines are plotted as a visual aid to guide the reader in observing how the higher‐order modes modulate the fundamental HPhP mode.

In an effort to investigate the isotope influence upon the phonon damping in α‐MoO_3_, we perform near‐field optical measurements of HPhPs, which are highly sensitive to their respective TO phonons to which they are coupled. We employ scattering‐type scanning near‐field optical microscopy (s‐SNOM; see methods) of HPhPs supported in mechanically exfoliated isotopic flakes across the three MIR RBs. In this work, we utilize two different illumination sources. For the RB_1_, the s‐SNOM system is coupled with a tunable free‐electron laser (FEL) to access the low frequencies not typically available in tunable table‐top light sources [54, 55]. The remaining RB_2_ and RB_3_ were probed using a tunable quantum cascade laser (QCL) as the illumination source to excite the respective in‐plane hyperbolic and elliptic HPhPs. To understand the frequency dependence of the HPhPs probed in this work, we consider the free‐space normalized biaxial analytical dispersion relation [15, 56]

(3) $$\frac{k}{k_{o}}=\frac{\rho }{k_{o}d}\left[\tan^{-1}\left(\frac{\rho \varepsilon_{a}}{\varepsilon_{z}}\right)+\tan^{-1}\left(\frac{\rho \varepsilon_{s}}{\varepsilon_{z}}\right)+\pi l\right],l=0,1,2\text{…}$$

and

(4) $$\rho =i\sqrt{\frac{\varepsilon_{z}}{\varepsilon_{x}\cos^{2}\left(\alpha \right)+\varepsilon_{y}\sin^{2}\left(\alpha \right)}}$$

where *k* is the real‐valued in‐plane HPhP wavevector, *k*_0_ is the free space wavevector, *d* is the flake thickness, *l* is the discrete HPhP mode order, ε_*a*_ is the permittivity of air, ε_*s*_ is the permittivity of our substrate, and ε_*x*,*y*, *z*_ are the respective in‐ and out‐of‐plane permittivities of the α‐MoO_3_. The factor ρ includes the angle α between the x ([100]) direction and the in‐plane incident excitation source wavevector‐ where α is fixed to $0^{\circ }$ when measuring along the [100] and $90^{\circ }$ along the [001]. The unitless polariton wavevector, $\frac{k}{k_{o}}$, is normalized by the free space wavevector, and we only consider propagating modes ($\frac{k}{k_{o}}$ > 1) where the HPhPs subside. s‐SNOM images of HPhPs excited at similar real permittivity values in ^98^MoO_3_ at 920 cm^−1^ (ε_*r*_ =  −2.22) and Mo^18^O_3_ (ε_*r*_ = −2.66) and ^98^Mo^18^O_3_ (ε_*r*_ = −2.71) at 880 cm^−1^ are shown in Figure 2a–c, respectively. As predicted from far‐field measurements, we observe a ∼40 cm^−1^ red shift in the RBs from ^18^O enrichment since the s‐SNOM images are near‐identical for the offset excitation frequencies. This spectral shift is more apparent in the near‐field by extracting the line profiles of these HPhPs supported in similar thickness (∼200 nm) flakes of similar polariton wavelengths (λ_*p*_) at different excitation frequencies (Figure S7.1 for specific AFM details). The resulting line profiles from Figure 2a–c plotted in Figure 2d display a consistent $\lambda_{p}∼0.5$ µm and a 40 cm^−1^ red shift from the ^98^MoO_3_ at 920 cm^−1^ to the Mo^18^O_3_ and ^98^Mo^18^O_3_ at 880 cm^−1^, respectively. In all cases, both tip $\left(\frac{\lambda_{p}}{2}\right)$ and edge‐launched (λ_*p*_) HPhPs are present due to the orientation of the flake edge set to be normal to the in‐plane polarization of the incident light.

Due to the possible presence of higher‐order modes in the s‐SNOM measurements, we perform all our polariton analyses in momentum space. Recent metrology has been developed by *Yuan* et al. to accurately extract the complex HPhP wavevector along different angles of the concave wavefront [57]. However, our edge‐launched HPhPs lack the necessary concave wavefront to extract multiple line profiles for such an approach. Thus, we approach our HPhP wavevector extraction with the more traditional approach the edge‐launched wave. Therefore, we employed an FFT analysis (see Section S8 for more details) to separate the HPhP modes in momentum space and extract their real (Re(*k*)) and imaginary (Im(*k*)) wavevectors. The HPhPs propagating in ^98^MoO_3_ at 880 cm^−1^ in Figure 2e further illustrates the 40 cm^−1^ red shift when compared to the s‐SNOM images of the Mo^18^O_3_ and ^98^Mo^18^O_3_ HPhPs in Figure 2b,c, excited at the same frequency. In addition to the comparisons of the polaritonic wavelengths outlined above, from the line scans provided, additional frequency components are apparent. These additional frequencies are clearly visible in the line profile plotted in Figure 2f. We identify the higher‐order modes present, through a fast‐Fourier transform (FFT) bandpass filter on the raw line profile to separate any noise, far‐field signal, and separate the higher‐order modes present in momentum space (see Section S8 for more details). The higher‐order modes are designated by each distinct Fourier peak with increasing momentum. The bandpass regions are then replotted in Figure 2f in the respective dotted and dashed lines. It is evident that up to the possible third‐order (*l* = 2) mode is present within the measured line profiles of the isotopically enriched samples. The influence of the third‐order mode is primarily seen in the first fringe of the first‐order (*l* = 0) mode, where the single peak is split into a doublet due to the first two fringes of the third‐order occurring before the mode is estimated to decay. The second‐order (*l* = 1) mode propagates further from the edge, resulting in additional fringes and shoulders of the first‐order mode. The presence of higher frequency components associated with higher‐order HPhP modes are a clear result of the reduced phonon linewidths (increased phonon lifetimes) through isotopic enrichment akin to what was reported by *Giles* et al. in their study of HPhPs in isotopically enriched hBN [42]. While higher‐order HPhPs intrinsically have much larger momenta than the fundamental mode, the redshift in the HPhP dispersion from ^18^O enrichment broadens the confinement capabilities of α‐MoO_3_ toward lower excitation frequencies. Now alongside hBN, we establish that isotopically enriched α‐MoO_3_ can support highly confined modes through the direct excitation of higher‐order HPhPs. Prior observations of higher‐order modes in α‐MoO_3_ were only able to be realized by scattering incident light from a 3C‐SiC nanowire [6] or by suppression of the fundamental mode using substrates with negative real permittivity, such as 4H‐SiC or Au [58]. Recent efforts have achieved efficient excitation of higher‐order modes in α‐MoO_3_ through the implementation of a secondary scattering event [59]. Thus, this represents a direct observation of propagating, higher order modes within isotopically enriched MoO_3_.

**FIGURE 3:**
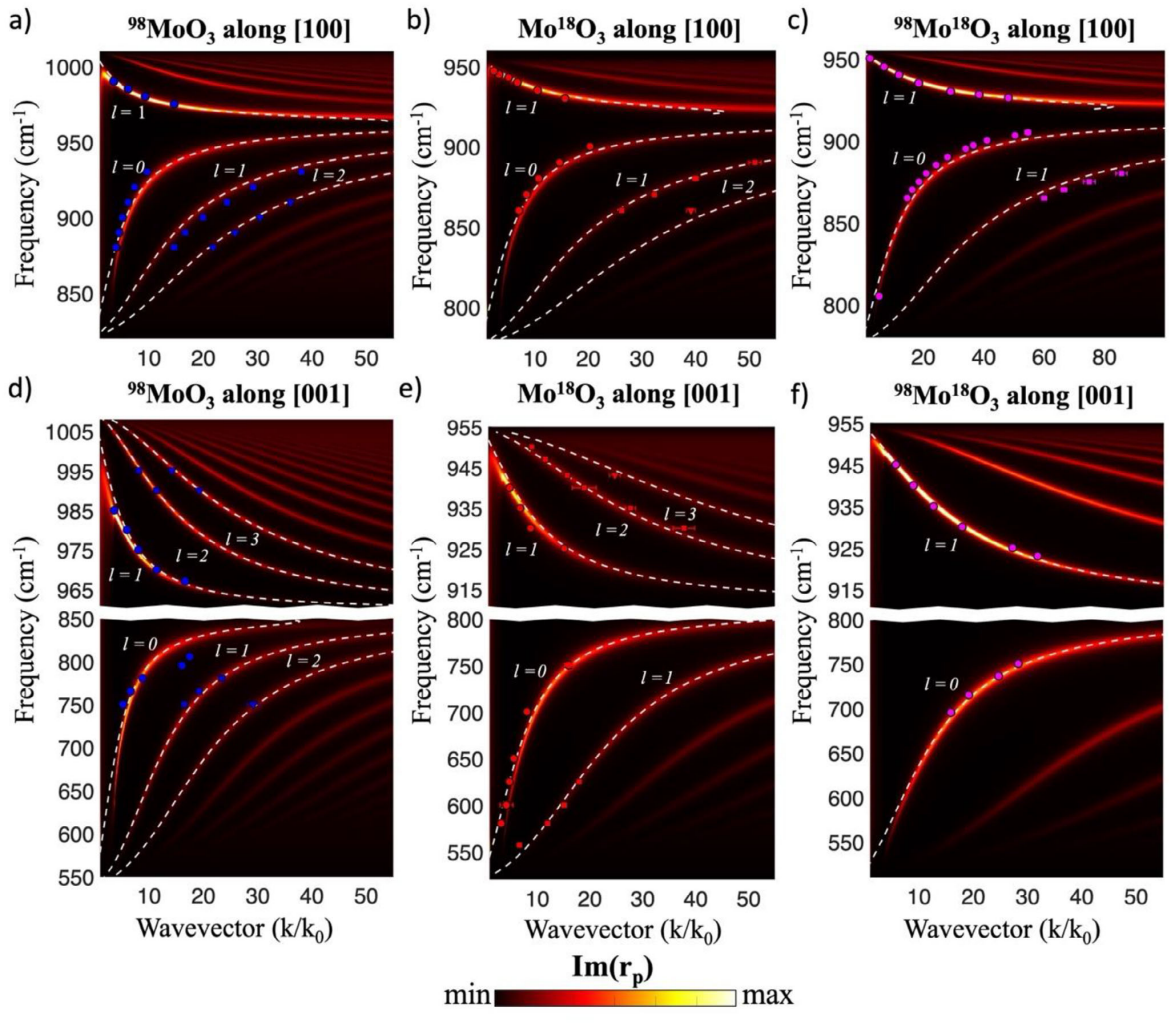
Experimental mapping the HPhP dispersion in different isotopically enriched α‐MoO_3_. (a–c) TMM calculations of p‐polarized imaginary‐part of the reflection coefficient (Im(r_p_)) along the [100] for ^98^MoO_3_, Mo^18^O_3_, and ^98^Mo^18^O_3_, respectively. (d–f) Similar TMM calculations and experimental data along the [001] for the same samples. The graphs were truncated in frequency gap between the RB_1_ and RB_3_ and rescaled so that the RB_3_ is more visible to the reader. The analytical dispersion from Equation (3) is calculated for each isotopic variation and plotted as a white dashed line for all the experimentally observed orders. Data points extracted from s‐SNOM images for each sample are overlayed in their respective plots. The error bars are propagated from the FFT analysis and laser linewidths.

Using the approximate isotopic dielectric functions, we calculated the imaginary part of the reflection coefficient, Im(*r_p_*), with the transfer matrix method (TMM) [60] for an air/α‐MoO_3_/Si structure along both in‐plane crystallographic directions. The analytical hyperbolic dispersions for ^98^MoO_3_, Mo^18^O_3_, and ^98^Mo^18^O_3_ are overlayed on the calculated Im(*r_p_*) values and alongside the experimental Re(*k*) values of HPhPs propagating along the [100] ([001]) axis in Figure 3a–f. We provide the dispersion mapping for different sample thicknesses in Figure S9.1. We see excellent agreement in all samples between the extracted HPhP momenta and their analytical dispersions. The higher‐order modes discussed in Figure 2f and present in other excitation frequencies are extracted from the FFT analysis are validated as second and third‐order modes. Due to the spectral redshift from ^18^O enrichment, we have limited access in frequency where we can probe the third‐order modes without significant damping. Thus, we demonstrate real‐space imaging of reduced loss, spectrally shifted HPhPs in isotopically enriched α‐MoO_3_. Furthermore, this lower scattering loss enables the excitation and imaging of higher‐order HPhP modes without the aid of a sub‐diffractional scattering source on the sample.

In contrast to the in‐plane hyperbolic bands, the in‐plane elliptical RB_3_ in both ^98^MoO_3_ and Mo^18^O_3_ only supported higher‐order modes along the [001] direction. In general, the narrow bandwidth of the RB_3_ compared to RB_1‐2_ result in slower HPhP group velocities $\left(v_{g}=\frac{\partial \omega }{\partial k}\;\right)$. It is clear from Figure 3 that the group velocities of the RB_3_ are much slower along the [100] than the [001] due to the dispersion asymptotic limits for the RB_3_ *l*  = *−1* branch in ^98^MoO_3_ it is roughly 961 cm^−1^ along the [001] but 971 cm^−1^ along the [100]. As discussed by *Sternbach* et al. in SI note 12, the spectral bandwidth of the light source imposes additional scattering losses via the excitation of similar momenta polaritons that destructively interfere with one another [61]. In a similar case, a larger range of HPhPs are excited for a slower group velocity. As a result, higher‐order modes for all isotopic samples along the [100] suffer more scattering losses compared to those along the [001]. The analytical hyperbolic dispersion, see Equation (3), contains a thickness dependence which will result in a slower group velocity for thinner flakes. We note that the ^98^Mo^18^O_3_ group velocities of the higher‐order modes shown in Figure 3f are slower than the other two isotopic samples due to the large discrepancy in thickness. A study on the HPhP group velocity would serve as a baseline requirement for efficient excitation of higher‐order modes but is beyond the scope of this work.

The parameters for the dielectric function describing the dispersive nature for each isotopically enriched α‐MoO_3_, which have not been previously investigated elsewhere, are provided here. After verifying the spectral shifts in the near‐field regime, we report the approximate experimental Lorentz oscillator parameters listed in Table 1 for the isotopic α‐MoO_3_ crystals in this work and a comparison to previously published isotopically enriched α‐MoO_3_ values provided in Table S10.1. Aside from the damping parameter, the TO and LO frequencies and high‐frequency dielectric constants were slightly tuned in the TMM calculations to fit with the experimental dispersion points in Figure 3.

**TABLE 1:** Experimental dielectric function parameters of isotopically enriched α‐MoO_3_.

Isotope	$\omega_{x}^{TO}$ (cm^−1^)	$\omega_{x}^{LO}$ (cm^−1^)	$\varepsilon_{x}^{\infty }$	$\omega_{y}^{TO}$ (cm^−1^)	$\omega_{y}^{LO}$ (cm^−1^)	$\varepsilon_{y}^{\infty }$	$\omega_{z}^{TO}$ (cm^−1^)	$\omega_{z}^{LO}$ (cm^−1^)	$\varepsilon_{z}^{\infty }$
**^98^MoO_3_**	824	966	4.3	544	849	7.9	960	1009	2.0
**Mo^18^O_3_**	779	923	5.8	526	806	5.1	912	954	4.5
**^98^Mo^18^O_3_**	779	923	5.8	526	806	6.1	910	954	3.6

**FIGURE 4:**
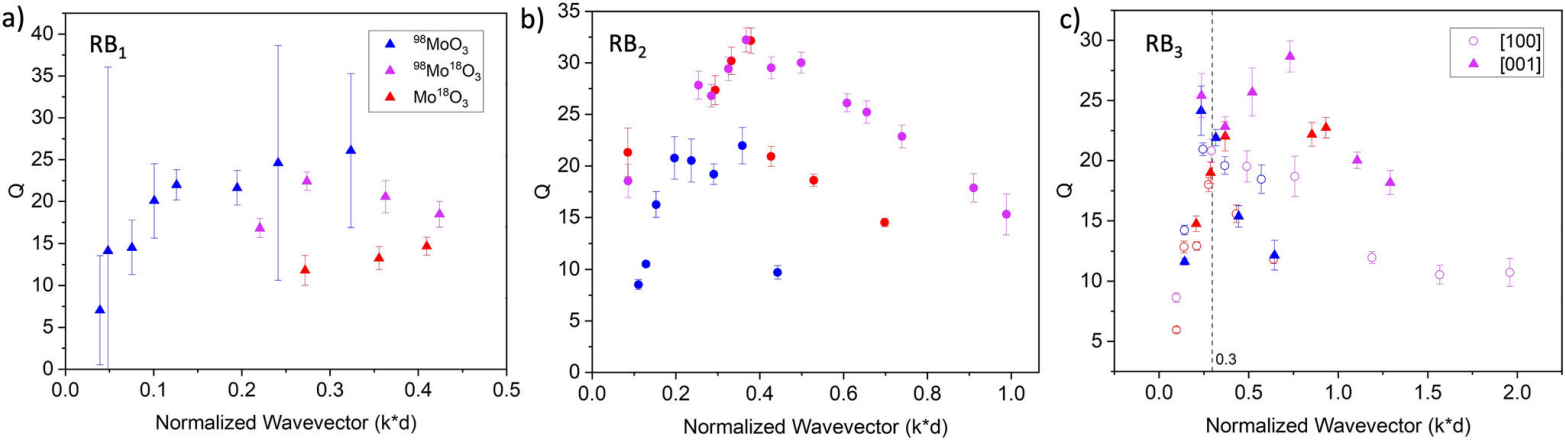
Isotopic α‐MoO_3_ polariton *Q*‐factors across the MIR Reststrahlen bands. (a–c) Polariton *Q‐*factors in the RB_1_, RB_2_, and RB_3_, respectively. All flakes measured for the results shown in Figures a and b are comparable in thickness (∼200 nm). In Figure 4c, ^98^MoO_3_ and Mo^18^O_3_ are 460 and 440 nm thick, while ^98^Mo^18^O_3_ is 200 nm. The circular data points depict the *Q*‐factors extracted along the [100], while the triangular data points show *Q* extracted along the [001] direction. The vertical dashed line in Figure 4c is plotted as a visual aid to indicate the wavevector vicinity where the RB_3_ anisotropic *Q*‐factor splitting occurs.

To investigate the optical loss across our isotopically enriched samples, we report the polariton *Q*‐factors for in all three RBs‐ defined by *Q* =2πRe(k)Im(k). From the complex polariton wavevector extracted from the FFT analysis, we calculate the Q‐factors as a function of the thickness normalized wavevector (k*d) across all isotopic samples for each RB_1‐3_ in Figure 4a–c, respectively, (larger sample thickness HPhP *Q*‐factors provided in Figure S11.1). This thickness‐normalized wavevector is easily obtained from manipulating Equation (1) and discarding the free‐space normalization. It is necessary to implement this unitless independent variable to compare HPhP *Q*‐factors across samples varying in thickness, as the momentum *k* scales inversely with the thickness *d*. This methodology has previously been employed by *Schultz* et al. to compare dispersions of different ^x^Mo isotopes in α‐MoO_3_ [46]. Within the RB_1_, we observe similar magnitudes of *Q* independent of which isotope was enriched (Figure 4a). Within this spectral region, we also measured naturally abundant α‐MoO_3_ and report its HPhP *Q*‐factors (Figure S11.2). Importantly, however, using an FEL as an excitation source can lead to inconsistent *Q*‐factors across isotopic samples due to the variable spectral bandwidth and signal‐to‐noise ratio (SNR) over a large frequency range, such as the excitation frequencies chosen for the RB_1_ (550–820 cm^−1^). Likewise, detectors at lower frequencies operate with a lower sensitivity. Therefore, HPhPs of similar momenta will have different experimental excitation conditions when measured at different frequencies, which is unavoidable due to the red shift from oxygen enrichment. Aside from varying experimental conditions, recall from Figure 1c that the RB_1_ TO is still significantly more damped than the other two corresponding TO phonons in RB_2_ and RB_3_. While we claim narrower linewidths from oxygen enrichment in the far‐field and believe similar enhancements would be present in the near‐field, our current measurements do not validate such an assumption. On the other hand, we observe a ∼50% increase in *Q*‐factor maxima within the RB_2_ for both Mo^18^O_3_ and ^98^Mo^18^O_3_ with respect to ^98^MoO_3_ and a slight shift in normalized wavevector where the *Q*‐factor is maximized (Figure 4b). Here, the *Q*‐factor maxima increase is due to the moderate increase in phonon lifetimes from ^18^O enrichment, as previously discussed. We also lend this *Q*‐factor enhancement to a slight increase in free‐space wavevector compression. The confinement factor ($\frac{k}{k_{0}}$) for the thickness‐normalized wavevector is equal in both Mo^18^O_3_ and ^98^Mo^18^O_3_ yet greater than in ^98^MoO_3_ (Figure S12.1). This is a result of the significant red shift due to ^18^O enrichment, where the HPhPs carry the same wavevector for a reduced free‐space wavevector. The *Q*‐factor maximizes at the wavevector where both the TO absorption and HPhP scattering rates are minimized – lower wavevectors are dominated by absorption loss (γ_α_) and higher wavevectors are dominated by scattering losses. Knighton et al. discusses the relation between phonon polariton dispersion and damping rate, where they show increasing polariton damping rates (γ_*p*_) for increasing polariton wavevectors [62]. As a result, the polariton damping rate increases with wavevector as the HPhP branch approaches the asymptotic limit. Therefore, the increased scattering from the Mo isotopic disorder (γ_*Mo*_) shares the same relationship as the damping rates due to the negligible change in *Q*‐factor at low wavevector and the ∼50% increase in *Q* across the higher wavevectors from Mo^18^O_3_ to ^98^Mo^18^O_3_. In comparison to prior works of Mo isotopic enrichment in α‐MoO_3_ [45, 46], we emphasize that the enhancement in *Q*‐factor from Mo^18^O_3_ to ^98^Mo^18^O_3_ is not simply an additive enhancement but more so a momentum‐dependent enhancement between the lower optical lifetimes (lower momenta) and reduced Mo isotopic disorder scattering loss (higher momenta). As a result of dual isotopic enrichment, HPhPs in ^98^Mo^18^O_3_ exhibit a higher *Q*‐factor across a broader range of momenta in the RB_2_. Similar to the thermal control over polaritonic topological states in surface HPhPs supported in YVO_4_ crystals [63], we believe that thermal wavefront shaping is an additional tuning parameter now available for high *Q*‐factor HPhPs in isotopically enriched α‐MoO_3_.

Lastly, we expect a high sensitivity to the change of *Q*‐factors along both directions of the RB_3_, due to the ultra‐narrow linewidth of the respective TO phonon. The elliptic behavior of the RB_3_ is demonstrated through the distinct *Q*‐factors of HPhPs propagating along the [100] or [001] (Figure 4c; Figure S13.1 for more clarity). Zhao et al. reports similar *Q*‐factors for both ^92^MoO_3_ and ^100^MoO_3_ along the [100] [45] compared to the ^98^MoO_3_ *Q*‐factors we report in this work. These findings corroborate the understanding that any Mo enrichment reduces the scattering loss due to isotopic disorder while the choice of isotope has minimal influence on the *Q*‐factor maxima due to the small mass variance. In the large momenta regime (k*d > 0.3), we observe higher *Q*‐factors in ^98^MoO_3_ along the [100] than the [001]. This anisotropic relation of *Q*‐factors in the RB_3_ is due proximity of the RB_3_ TO phonon (961 cm^−1^) to the frequency where the *l* = 0 branch approaches the asymptotic limit; ∼961 cm^−1^ along the [001] and ∼971 cm^−1^ along the [100]. Therefore, the differences in HPhP dispersion impose a larger γ_α_ on HPhPs along the [001] than the [100]—leading to higher *Q*‐factors along the [100]. Surprisingly, we observe a stronger and inverted anisotropic response from ^18^O enrichment, where *Q*‐factors are now greater along the [001] than along the [100]. The larger *Q*‐factors along the [001] than along the [100] implies that γ_α_ is now considerably less than γ_*p*_‐ causing the in‐plane axis of higher *Q* HPhPs to flip from [100] to [001]. This observation further supports the modest RB_3_ TO lifetime improvements that we expect from ^18^O enrichment. Lastly, we correlate these improvements to the ∼100% enhancement of the *Q*‐factor maxima along the [001] in Mo^18^O_3_ with respect to ^98^MoO_3_. We do not compare the *Q*‐factors in ^98^Mo^18^O_3_ to the other two isotopes due to a large discrepancy in sample thickness that the unitless wavevector cannot account for. However, we still expect similar enhancements in *Q*‐factor maxima from ^18^O enrichment and the broadest wavevector range of high‐*Q* HPhPs to be supported in thin flakes of ^98^Mo^18^O_3_.

## 3 Outlook

In this work, we investigated both avenues of optical and acoustic phonon atomic dependencies through far‐field and near‐field optical measurements and thermal conductivity measurements in isotopically enriched α‐MoO_3_. We provide an in‐depth theoretical analysis that both validates and predicts various phonon scattering mechanisms present in α‐MoO_3_. Raman and FTIR spectroscopy were performed to initially observe the expected ∼40 cm^−1^ redshift from ^18^O enrichment and linewidth reductions. Consistent with this observation, our mode‐resolved three‐phonon scattering analysis confirms that the lifetime enhancement is not captured by a simple mass‐scaling of scattering rates. Rather, it reveals that the ^18^O enrichment renormalizes the harmonic phonon spectrum and thereby reshapes the available three‐phonon phase‐space and coupling landscape, particularly those driven by oxygen oscillations, such that the net anharmonic linewidths decrease. We report the approximate dielectric functions of ^98^MoO_3,_ Mo^18^O_3_, and ^98^Mo^18^O_3_ from their respective phonon shifts. TDTR measurements were conducted across the same isotopes and validated with DFPT calculations. Further theoretical thermal analysis reveals the possibility to deterministically engineer directional thermal transport in isotopic α‐MoO_3_ flakes. The near‐field IR properties were probed with s‐SNOM to measure the HPhPs in each of the three MIR RBs. We experimentally report propagating higher order HPhP modes up to *l* = 2 without imposing additional scatterers. The highly anisotropic character of each TO phonon from their respective RBs is reflected into the degree of HPhP *Q*‐factor enhancements that are only observed due to isotopic enrichment. Through these efforts, we discuss the interchange of loss mechanisms at play in α‐MoO_3_ with respect to HPhP wavevector: predominantly being absorption and polariton damping rates. This work experimentally explores the intrinsic limit of tuning *Q*‐factors in α‐MoO_3_ through isotopic enrichment. We demonstrate the benefits from a dual‐element isotopic ^x^Mo^18^O_3_ slabs in supporting enhanced HPhP *Q*‐factors and higher wavelength compression than with ^16^O. Such a platform can have significant impact in the extension of current applications, such as advanced canalized HPhP imaging [64] and dispersion engineering [65]. In comparison with external tuning parameters such as substrate engineering, gating, or subdiffractional patterning, we establish intrinsic engineering of desired optical and thermal responses in dual‐element isotopic α‐MoO_3_, where the latter is not readily achieved compared to isotopic enrichment. This work also has a direct impact in expanding the already tunable platform of telluride molybdenum quaternary oxides demonstrated by Sun et al. [66] through implementation of the same dual‐element enrichment strategy of ^x^Mo and ^18^O and the modal hybridization with HPhPs in ^x^Mo^18^O_3_. Further work with isotopically enriched α‐MoO_3_ could serve as an ideal candidate to experimentally discern the contributions of optical modes to thermal transport, which is conventionally an acoustic dominated response. In the context of optic phonons and optically active modes, future investigations should explore the impact of tunable phonon linewidths from mixed ^16^O/^18^O ratios upon modal hybridization and strong coupling in isotopic heterostructures.

## 4 Methods

### 4.1 Sample Growth and Preparation

The α‐MoO_3_ crystals were primarily grown using a reactive vapor transport method that is detailed in a companion report [67]. In short, a molybdenum (naturally abundant or isotope‐enriched) metal source is heated in an inert environment within a multi‐zone tube furnace, where it is rapidly oxidized and vaporized by O_2_ flow of select isotope enrichment. Numerous mm‐ to cm‐scale α‐MoO_3_ crystals were grown after the precursor vapor condenses on the walls of the tube within the cooler furnace zone. The ^98^MoO_3_ samples were grown using a similar non‐reactive vapor transport method using ^98^Mo‐enriched MoO_3_ powder (IsoFlex USA, 98.42% enrichment) as the evaporating source and *N*_2_ carrier gas. The free‐standing crystals were then exfoliated with tape and transferred to silicon substrates until the desirable surface area and thicknesses were achieved for both far and near‐field measurements.

### 4.2 Density Functional Perturbation Theory Calculations

First‐principles density functional perturbation theory (DFPT) calculations were performed using Quantum ESPRESSO [68] to compute interatomic force constants (IFCs) and three‐phonon scattering rates for layered α‐MoO_3_. The van der Waals density functional vdW‐DF‐optB88 (vdw‐df‐obk8) [69] was used to accurately capture the weak interlayer interaction following previous validation for MoO_3_ [70]. Optimized norm‐conserving Vanderbilt (ONCV) pseudopotentials were used for Mo and O, with a plane‐wave kinetic energy cutoff of 120 Ry [71]. Self‐consistent field calculations were converged to a threshold of 10^−^^10^ Ry.

Harmonic (second‐order) IFCs were calculated using the finite displacement method in supercells commensurate with the desired phonon wavevector mesh. For the 3 × 3 × 1 supercell (144 atoms), a Γ‐centered 4 × 4 × 3 k‐point mesh was employed with 16 symmetry‐inequivalent displacement configurations. The 3 × 3 × 2 supercell (288 atoms) used a 4 × 4 × 2 k‐point mesh with 24 configurations. These IFCs were used to construct dynamical matrices and compute phonon dispersions.

Anharmonic (third‐order) IFCs, which govern three‐phonon scattering processes, were computed using a 2 × 2 × 1 supercell (64 atoms) with a denser 6 × 6 × 3 k‐point mesh. A total of 6,680 symmetry‐inequivalent configurations were required to fully sample the third‐order force constant tensor. Non‐analytic corrections (NAC) to the dynamical matrix, arising from long‐range dipole–dipole interactions, were included using Born effective charge tensors and the high‐frequency dielectric tensor obtained from density‐functional perturbation theory as implemented in Quantum ESPRESSO *ph.x*. The NAC was evaluated along the principal crystallographic directions [100], [010], and [001], enabling resolution of the polarization‐dependent LO–TO splitting for infrared‐active modes at the Brillouin zone center.

### 4.3 First‐Principles Three‐Phonon Scattering and Linewidth Calculations

Phonon dispersions, three‐phonon scattering rates, and the lattice thermal conductivity were computed using the phono3py package [72], with the harmonic and anharmonic IFCs from the finite‐displacement DFPT calculations as input.

Non‐analytic corrections to the dynamical matrices were included in phono3py using the Born effective charges and dielectric tensor, following the Gonze and Lee formalism [73]. This treatment captures LO–TO splitting and polar‐optical phonon behavior. The resulting phonon frequencies and eigenvectors, including NAC, were then used to evaluate three‐phonon scattering processes.

Anharmonic phonon linewidths arising from three‐phonon scattering were computed within the single‐mode relaxation time approximation (SMRT) as implemented in phono3py, by evaluating the imaginary part of the phonon self‐energy from all energy‐ and momentum‐conserving three‐phonon processes. Scattering rates were obtained from the third‐order IFCs and phonon eigenvectors following the formalism of Togo, Chaput, and Tanaka [72]. Γ‐point phonon linewidths were converged using a 36 × 36 × 12 Γ‐centered q‐point mesh at 300 K. Systematic tests of convergence verified this mesh density to be sufficient for the modes of interest up to the accuracy desired.

Isotope disorder scattering was included using the Tamura model [74]. The phonon–isotope scattering rate depends on the mass variance parameter:

$$g=\sum_{i}f_{i}{\left(\frac{m_{i}}{\bar{m}-1}\right)}^{2}$$

where *f_i_* and *m_i_* are the fractional abundance and mass of isotope i, and m̄ is the average atomic mass. For natural‐abundance α‐MoO_3_, *g* was evaluated including all stable Mo isotopes (^92^Mo–^100^Mo) and O isotopes (^16^O, ^17^O, ^18^O). For the isotopically enriched samples, *g* was recomputed assuming 98% ^98^Mo and 75%–100% 18O enrichment, consistent with the experimental isotopic purities.  In addition to modifying the mass variance parameter, the atomic masses used in constructing the dynamical matrix were adjusted to reflect the isotopic composition of each sample. Since the interatomic force constants are mass‐independent, the same IFCs were used for all isotopic compositions, but the phonon frequencies and eigenvectors were recalculated using the isotope‐averaged atomic masses. This captures the intrinsic frequency shifts and modified group velocities arising from isotopic mass substitution, which are distinct from the isotope‐scattering effects encoded in *g*. The total scattering rate for each mode was taken as the sum of the anharmonic three‐phonon and isotope‐disorder contributions, Γ_total_ = Γ_anharmonic_ + Γ_iso_, and the phonon lifetime was defined as τ = (2 Γ_total_)^−^^1^.

The lattice thermal conductivity tensor κ was calculated at 300 K within the relaxation time approximation (RTA) by solving the linearized phonon Boltzmann transport equation using phono3py. A 36 × 36 × 12 Γ‐centered q‐mesh was used to sample the Brillouin zone. In this framework, κ_αβ_ is calculated as:

$$\;\kappa_{\alpha \beta }=\left(\frac{1}{VN_{q}}\;\right)\Sigma_{q,s}\;C_{qs}\;v_{qs,\alpha }⊗v_{qs,\beta }\;\tau_{qs}$$

where *V* is the crystal volume and *N*_q_ is the number of q‐points, and the sum runs over wavevectors q and phonon branches s. The mode heat capacity *C*_qs_ and group velocity components *v*_qs,α_ are computed from the harmonic IFCs, while τ_qs_ is the phonon lifetime determined by the total scattering rate.

### 4.4 Raman Spectroscopy

Raman spectroscopy was performed using a LabRAM HR Raman microscope (Horiba) equipped with a 532 nm excitation laser and a 1800 gr/mm grating. Because of the polarization‐dependent biaxial anisotropy of α‐MoO_3_, a consistent azimuthal orientation is required to achieve reproducible Raman peak intensity ratios [75]. Therefore, the α‐MoO_3_ flakes were supported on *c*‐axis‐oriented sapphire substrates, and the [001] direction oriented parallel to the horizon of the instrument microscope. The spectra were normalized to the intensity of the strongest α‐MoO_3_ peak (819 cm^−1^ for unenriched crystals) and the *x*‐axis calibrated using the Rayleigh peak at 0 cm^−1^. The instrument resolution, measured as the full width at half maximum (FWHM) of the Rayleigh scattering peak, was 0.8 cm^−1^. The Raman linewidths were collected by averaging five measurements fitted with Lorentzian peak shapes.

### 4.5 FTIR Spectroscopy

The FTIR reflection measurements were performed through a Bruker Hyperion 2000 microscope equipped with the Bruker 15x Grazing Angle Objective (GAO) coupled to a Bruker Vertex70v FTIR spectrometer and collected with a broadband MCT detector (400–8000 cm^−1^). Both a ZnSe beam splitter and Polyethylene linear polarizer (∼600–5000 cm^−1^) were used to obtain polarized reflection spectra of isotopically enriched α‐MoO_3_. The 15x GAO was employed for its viewing mode with near normal incidence of ∼ 4°, where s and p polarization states nearly independently stimulate each in‐plane direction. When the 15x GAO is switched to its grazing mode (∼ 86°), we maintain a p‐polarization state and instead, rotate the crystal to excite each in‐plane direction. Internal microscope apertures of 50 × 50 µm were used to ensure light is collected from the crystal. All spectra were collected with a 2 cm^−1^ spectral resolution and normalized with the spectra from a gold mirror taken with the same experimental conditions.

### 4.6 Near‐Field Characterization

The study of highly confined polaritons requires optical measurements with subdiffractional resolution, which can be achieved via s‐SNOM measurements [16]. In s‐SNOM, light illuminates and is scattered by a metal coated AFM tip; the scattered light is subsequentially detected by a suitable optical detector. The AFM tip acts as an antenna, allowing for simultaneous access and scattering of the near‐field from the sample directly below the tip, with a spatial resolution corresponding to the tip radius (typically ∼20 nm) [76, 77]. Additionally, the highly confined field at the tip apex provides enough momentum to couple to HPhPs [78]. The tip simultaneously scatters the near‐field from propagating polaritons that were either tip launched and edge reflected or directly edge launched. For the optical near‐field measurements presented in this publication, two commercial s‐SNOM devices by neaspec (Attocube GmbH) were utilized, coupled to two different light sources. Measurements in RB_2_ and RB_3_ were performed using a tunable quantum cascade laser (QCL; MIRCAT by Daylight Solutions). For measurements using this light source, an interferometric setup with an oscillating mirror in the reference arm (pseudo‐heterodyne detection scheme) was employed, allowing for a suppression of the far‐field and the separation of the optical amplitude and phase [79]. Measurements in RB_1_ were instead performed utilizing the tunable free‐electron laser FELBE located at the Helmholtz‐Zentrum Dresden‐Rossendorf (HZDR), which allows for accessing the required THz frequencies [17, 54, 55, 80]. The corresponding setup uses the self‐homodyne detection scheme instead due to the challenges in achieving pseudo‐heterodyne detection using a FEL. As the wavelength of the far‐field is roughly an order of magnitude longer than that of the measured polaritons, a separation of the two phenomena is possible in post‐processing [17].

## Author Contributions

**Ryan W. Spangler**, **Jon‐Paul Maria**, and **Joshua D. Caldwell** conceived the idea. **Ryan W. Spangler** grew the bulk isotopically enriched α‐MoO_3_ crystals under supervision of **Jon‐Paul Maria**. **Johnathan D. Georgaras** and **Jonah B. Haber** executed all the DFPT calculations and analysis under supervision of **Felipe Jornada**. **Ryan W. Spangler** performed all the Raman measurements and fittings supervised by **Jon‐Paul Maria**. **Daniel Hirt** performed the TDTR measurements under the supervision of **Patrick E. Hopkins**. **Thiago S. Arnaud** performed all the sample preparations for FTIR and SNOM measurements. **Thiago S. Arnaud** performed the FTIR reflection measurements supervised by **Joshua D. Caldwell**. **Thiago S. Arnaud** performed the majority of the SNOM measurements for the RB_2_ and RB_3_ across all isotopes, and **Maximilian Obst** performed the RB_3_ measurements for the thicker Mo^18^O_3_ sample; supervised by **Joshua D. Caldwell**. **Maximilian Obst**, **Gonzalo Álvarez‐Pérez**, **Mackey Long**, **Felix G. Kaps**, **Jakob Wetzel**, **Courtney Ragle**, **John E. Buchner**, **Youngji Kim**, **Aditha S. Senarath**, **Richarda Niemann**, **Giulia Carini**, **Unai Arregui‐Leon**, **Akash C. Behera**, **Ramachandra Bangari**, **Nihar Sahoo**, **Niels C. Brumby**, **J. Michael Klopf**, **Martin Wolf**, **Thomas G. Folland**, and **Alexander Paarmann** carried out the SNOM measurements at the Helmholtz‐Zentrum Dresden‐Rossendorf (HZDR) with the tunable free‐electron laser FELBE supervised by **Martin Wolf**, **Lukas M. Eng**, and **Susanne C. Kehr**. **Thiago S. Arnaud** and **Maximilian Obst** performed the HPhP momenta analysis from all the SNOM measurements supervised by **Joshua D. Caldwell**. **Thiago S. Arnaud**, **Ryan W. Spangler**, **Johnathan D. Georgaras**, **Jonah B. Haber**, and **Daniel Hirt** prepared the figures and drafted the manuscript with thorough feedback and revisions from **Maximilian Obst**, **Gonzalo Álvarez‐Pérez**, **Mackey Long**, **Mingze He**, **Unai Arregui‐Leon**, **Ramachandra Bangari**, **Susanne C. Kehr**, **Thomas G. Folland**, **Patrick E. Hopkins**, **Felipe Jornada**, **Jon‐Paul Maria**, and **Joshua D. Caldwell**.

## Conflicts of Interest

The authors declare no conflicts of interest.

## Supporting information




**Supporting File**: adma73629‐sup‐0001‐SuppMat.docx.

## Data Availability

All data collected and analyzed in this study is available upon request to the corresponding authors.
