# Supplementary material for: Tailoring Phonon‐Driven Responses in α‐MoO3 through Isotopic Enrichment
Source: Adv Mater. 2026 Jun 11;38(39):e73629. doi: 10.1002/adma.73629 (PMC13361168; doi:10.1002/adma.73629)
Supplement: Supplementary file 1 — Supporting File: adma73629‐sup‐0001‐SuppMat.docx. [file ADMA-38-e73629-s001.docx]

**Supplementary material: Tailoring phonon-driven responses in α-MoO_3_ through isotopic enrichment**

*Thiago S. Arnaud* *^1,2^, Ryan W. Spangler* *^3^, Johnathan D. Georgaras* *^4^, Jonah B. Haber* *^4^, Daniel Hirt* *^5^, Maximilian Obst* *^1,2,6,7^, Gonzalo Álvarez-Pérez* *^8,9^, Mackey Long III* *^1,2^, Felix G. Kaps* *^6,7^, Jakob Wetzel* *^6,7^, Courtney Ragle* *^1,2^, John E. Buchner* *^1,2^, Youngji Kim* *^2^, Aditha S. Senarath* *^1,2^, Richarda Niemann* *^2^, Mingze He* *^2^, Giulia Carini* *^9^, Unai Arregui-Leon* *^9,10^, Akash C. Behera* *^9^, Ramachandra Bangari* *^12^, Nihar Sahoo* *^12^, Niels C. Brumby* *^9^, J. Michael Klopf* *^11^, Martin Wolf* *^9^, Lukas M. Eng* *^6,7^, Susanne C. Kehr^6,7^, Thomas G. Folland* *^12^, Alexander Paarmann* *^9^, Patrick E. Hopkins* *^5,13,14^, Felipe Jornada* *^4^*, Jon-Paul Maria* *^3^*, Joshua D. Caldwell* *^1,2^**

*^1^ Interdisciplinary Material Science, Vanderbilt University, Nashville 37235, TN, USA*

*^2^ Department of Mechanical Engineering, Vanderbilt University, Nashville 37235, TN, USA*

*^3^ Department of Materials Science and Engineering, The Pennsylvania State University, University Park 16802, PA, USA*

*^4^* *Department of Materials Science and Engineering, Stanford University, Stanford, CA 94305, USA*

*^5^ Department of Mechanical and Aerospace Engineering, University of Virginia, Charlottesville, Virginia 22904, USA*

*^6^Institute of Applied Physics, TUD Dresden University of Technology, Dresden 01187, Germany*

*^7^Würzburg-Dresden Cluster of Excellence - EXC 2147 (ct.qmat), Dresden 01062, Germany*

*^8^Istituto Italiano di Tecnologia, Center for Biomolecular Nanotechnologies, Lecce, Italy*

*^9^Department of Physcial Chemistry, Fritz Haber Institute of the Max Planck Society, Faradayweg 4-6, 14195, Berlin, Germany*

*^10^Department of Physics, Politecnico di Milano, Piazza Leonardo da Vinci 32, 20133, Milan, Italy*

*^11^Institute of Radiation Physics, Helmholtz-Zentrum Dresden-Rossendorf, Dresden 01328, Germany*

*^12^Department of Physics and Astronomy, University of Iowa, Iowa City 52242, IA, USA*

*^13^*Department of Materials Science and Engineering, *University of Virginia*, Charlottesville, Virginia 22904, USA

*^14^*Department of Physics, *University of Virginia*, Charlottesville, Virginia 22904, USA

* Correspondence to: [josh.caldwell@vanderbilt.edu](mailto:josh.caldwell@vanderbilt.edu), [jpm133@psu.edu,](mailto:jpm133@psu.edu,) and [jornada@stanford.edu](mailto:jornada@stanford.edu)

**Table of Contents**

S1. *Ab Initio* optical and acoustic phonon properties

S2. *Ab Initio* Raman-active phonon modes and lifetimes

S3. FTIR measurements of isotopic α-MoO_3_ flakes

S4. *Ab Initio* IR-active phonon modes and linewidths

S5. TDTR measurements of isotopic α-MoO_3_ flakes

S6. *Ab Initio* thermal conductivity and isotope mass-variance effects

S7. s-SNOM sample images and AFM measurements

S8. FFT analysis of HPhPs in real and momentum space

S9. HPhP dispersion mapping for thicker isotopic α-MoO_3_ flakes

S10. Comparison in dielectric function with literature

S11. Additional HPhP Q-factor plots

S12. Free space confinement from ^18^O enrichment

S13. Individual RB_3_ Q-factors in isotopically enriched α-MoO_3_ flakes

**Section S1: *Ab Initio* optical and acoustic phonon properties**

**Figure S1.1:** First-principles calculations of the phonon dispersion in α-MoO_3_ (black) and α-Mo^18^O_3_ (red).

We first compute the first-principles harmonic phonon dispersion of α-MoO₃ for all isotope compositions by density-functional perturbation theory (implemented in Quantum Espresso). Crucially, we include non-analytic corrections (NAC) to the dynamical matrix to account for the macroscopic electric fields generated by polar vibrations. This correction captures the longitudinal optical–transverse optical (LO–TO) splitting at the -point which opens the Reststrahlen bands (RB_1-3_) essential to the hyperbolic optical behavior discussed in this work. **Figure S1.1** compares the full dispersion for naturally abundant α-MoO₃ (¹⁶O, black) and fully ¹⁸O-enriched α-MoO₃ (red). As expected for a lattice in which oxygen is the lighter sublattice, oxygen substitution primarily red-shifts the mid- and high-frequency optical branches, including the phonons forming the three MIR Reststrahlen bands (RB_1-3_), while leaving the acoustic branches and most low-frequency optical modes nearly unchanged. These trends are consistent with the red shifts of the Raman, IR, and HPhP resonances observed in the main text and set the baseline for the isotope-dependent Raman, IR, and thermal analyses presented in Sections S2, S4, and S6.


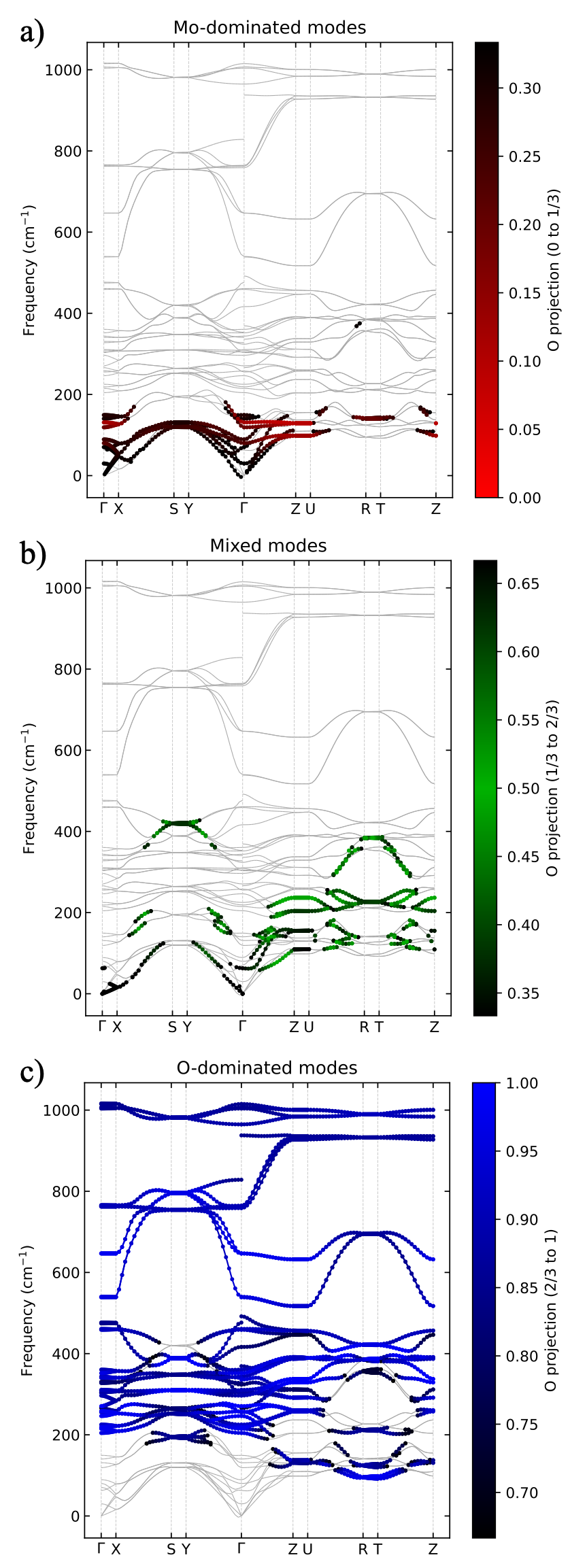


**Figure S1.2:** *Ab initio* atom-resolved phonon dispersion of α-MoO3. Phonon band structure along high-symmetry paths, decomposed by atomic character based on the squared eigenvector projections onto Mo and O sublattices. (Left) Mo-dominated modes, where the eigenvector projection onto oxygen atoms is less than 1/3, shown with a red color scale indicating Mo character (100 % = pure Mo motion). (Center) Mixed-character modes with intermediate O projection (1/3 to 2/3), displayed with a green color scale where maximum intensity (100 %) corresponds to equal Mo/O participation. (Right) O-dominated modes with O projection exceeding 2/3, shown in blue color scale indicating O character (100 % = pure O motion). Grey lines show all phonon bands for reference. In Figure S1.4 and Figure S1.8 we use ~ 180 cm⁻¹ as the threshold between acoustic and optical modes for analysis.

To quantify how different atoms participate in each phonon, we project the eigenvectors onto Mo and O sublattices and classify the modes as Mo-dominated, mixed, or O-dominated (**Figure S1.2**). The modes below ~ 200 cm⁻¹ are predominantly Mo-like and correspond to acoustic and low-energy optical branches that control heat transport, whereas the mid- to high-frequency optical modes above ~ 500 cm⁻¹ are strongly O-dominated and underlie the IR-active Reststrahlen bands and Raman-active stretching modes. Because the heat-carrying acoustic modes are dominated by the heavier Mo atoms while the HPhP-driving optical modes are dominated by the lighter O atoms, α-MoO_3_ offers a unique platform to decouple thermal and optical engineering via site-specific isotope selection. For the IR-active TO modes corresponding to the MIR RBs, we additionally visualize the real-space atomic translations in the primitive cell (**Figure S1.3**). As expected of the lighter mass driving the optical phonons, the oxygen atoms are the predominant drivers of the transverse oscillations. Due to the orthorhombic crystal structure of α-MoO_3_, the TO phonons along each crystallographic direction is dictated by the number of Mo atoms the oxygen is bonded to. The lowest energy TO phonon along the [001] belonging to the RB_1_ is dictated by the oxygen atom labeled as O(1), where it is bonded to three Mo atoms shown in **Figure S1.3a**. Subsequently, the RB_2_ and RB_3_ TO phonons are driven by oxygen atoms bonded to two and one Mo atom(s), respectively (**Figure S1.3b-d**). Taken together, these mode patterns and linewidth calculations suggest a qualitative correlation between the local constraint on the principal oxygen atom and the HPhP Q-factor: TO modes in which the relevant O site is more strongly bonded (and thus more restricted) exhibit smaller IR/Raman linewidths and, correspondingly, higher calculated Q-factors for the associated Reststrahlen band. In the following sections, we use first-principles calculations to confirm certain trends observed in experimental data. We calculate dispersions and mode characters to analyzing isotope-dependent Raman linewidths (Section S2), IR-active TO linewidths (Section S4), and lattice thermal conductivity and its decomposition into acoustic and optical contributions (Section S6).

**
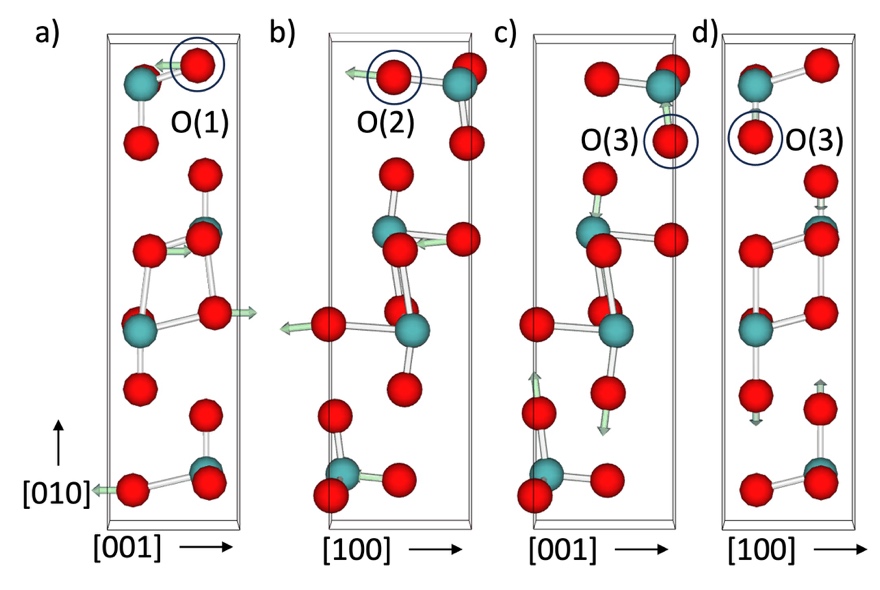
**

**Figure S1.3:** Visualized eigen displacements occurring for each TO phonon in a unit cell of α-MoO_3_ corresponding to the RB_1_ in a), RB_2_ in b), and RB_3_ in c) and d), respectively. The principal oxygen atom undergoing displacement is labeled in each of their respective figures.

**Section S2: *Ab Initio* Raman-active phonon modes and lifetimes**

The Raman shifts and linewidths extracted from the experimental spectra in Figure 1a are compared with the DFPT calculated values between equivalent isotopes in **Table S2.1-4**. The Raman spectra are dominated by three high-frequency modes, labeled B_1g_, A_g_, and B_2g_, which lie in the upper part of the optical manifold of the phonon dispersion (Figure S1.1). These modes sit well above the low-frequency Mo-dominated acoustic and optical branches and reside in the region where the eigenvectors are strongly oxygen-like, consistent with the large Raman intensities extracted from first-principles Raman calculations and from the experimental spectrum of natural α-MoO_3_. Using the atom-resolved phonon dispersion (Figure S1.2), we classify the experimentally observed Raman-active modes as predominantly O-dominated and further distinguish whether their displacements are mainly in-plane or out-of-plane, which is the key descriptor for how they couple to in-plane versus out-of-plane optical fields.

**Table S2.1:** Comparison of Raman shifts and linewidths between experimental and *ab initio* values in naturally abundant α-MoO_3_

**Table S2.2:** Comparison of Raman shifts and linewidths between experimental and *ab initio* values in ^98^MoO_3_

**Table S2.3:** Comparison of Raman shifts and linewidths between experimental and *ab initio* values in Mo^18^O_3_

**Table S2.4:** Comparison of Raman shifts and linewidths between experimental and *ab initio* values in ^98^Mo^18^O_3_

The subset of modes analyzed here was selected using the Raman implementation in Quantum ESPRESSO, which evaluates the derivative of the macroscopic dielectric tensor with respect to each normal mode within DFPT and from this, constructs mode-resolved Raman tensors and intensities. Modes with the largest calculated Raman activity and frequencies matching the experimental peaks were then used for detailed linewidth analysis. Within the single-mode relaxation-time approximation, the intrinsic anharmonic linewidth of a Raman-active mode $\lambda$ at wavevector $\Gamma$is obtained from the imaginary part of the phonon self-energy built from third-order interatomic force constants, which we evaluate using the standard three-phonon expression^[1]^:

| $\Gamma_{\lambda}\left( \omega\right)=\frac{18}{\hbar^{2}}\sum_{\lambda^{'},\lambda^{''}} \left\vert\Phi_{-\lambda\lambda^{'}\lambda^{''}} \right\vert^{2}\left[ \left( n_{\lambda^{'}}+ n_{\lambda^{''}}+1 \right)\delta\left( \omega-\omega_{\lambda^{'}}-\omega_{\lambda^{''}} \right)+\left( n_{\lambda^{'}}-n_{\lambda^{''}} \right)\left( \delta\left( \omega+\omega_{\lambda^{'}}-\omega_{\lambda^{''}} \right)-\delta(\omega-\omega_{\lambda^{'}}+\omega_{\lambda^{''}}) \right) \right]$ | [Eq.S2.1] |
| --- | --- |

where $\lambda\equiv\left( \mathbf{q},j \right)$labels a mode, $\Phi_{-\lambda\lambda^{'}\lambda^{''}}$ are the three-phonon interaction matrix elements, $\omega_{\lambda}$and $n_{\lambda}$are the phonon frequency and Bose–Einstein occupation, and the three δ–functions describe decay and absorption/emission processes; the total scattering rate is $1/\tau_{\lambda}=2\Gamma_{\lambda}$. This formalism follows standard treatments of third-order phonon–phonon scattering and is equivalent to the expressions implemented in phono3py and related BTE solvers.

Finally, the mode-resolved three-phonon scattering analysis in **Figure S2.1**, together with the summary statistics in **Tables S2.5** and **S2.6**, decomposes $\Gamma_{\lambda}$ for the three experimentally observed Raman modes in naturally abundant α-MoO_3_ into contributions from acoustic–acoustic (A+A), acoustic–optical (A+O), optical–acoustic (O+A), and optical–optical (O+O) channels and into different regions of the Brillouin zone via the histogram of $\mid\mathbf{q}\mid/\mid\mathbf{q}_{\mathrm{BZ}}\mid$. The corresponding maps of daughter-modes character — referring to the two modes other than the primary parent mode in the momentum- and energy-conserving scattering triplet — show that the linewidths of these modes are dominated by scattering into O-dominated daughters, with A+O and O+O processes providing the largest fraction of $\Gamma_{\lambda}$. The tabulated fractions make explicit that channels involving two O-like daughters (O–O region in Figure S2.1) account for most of the anharmonic broadening, consistent with the picture that ^18^O enrichment primarily modifies the phase-space and matrix elements of O-dominated decay pathways and thereby drives the systematic linewidth reductions discussed above and in the main text.

**Table S2.5:** Acoustic/optical decomposition of the three-phonon scattering channels for Raman-active modes.


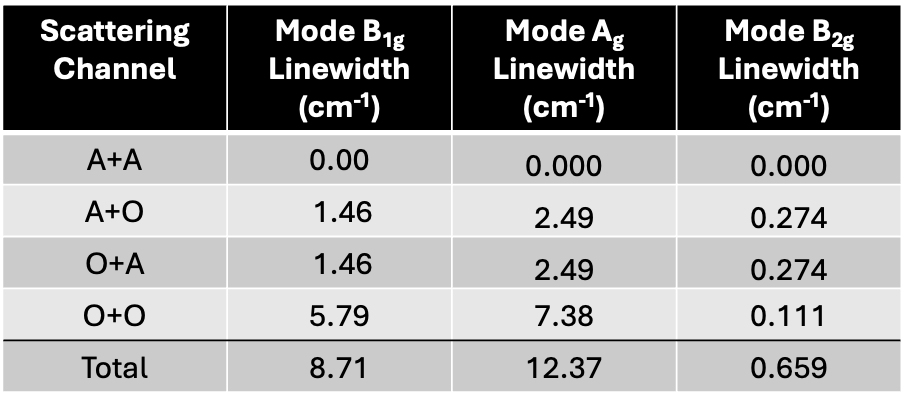


**Table S2.6:** Mo/O atomic-character decomposition of three-phonon scattering channels for Raman-active modes.


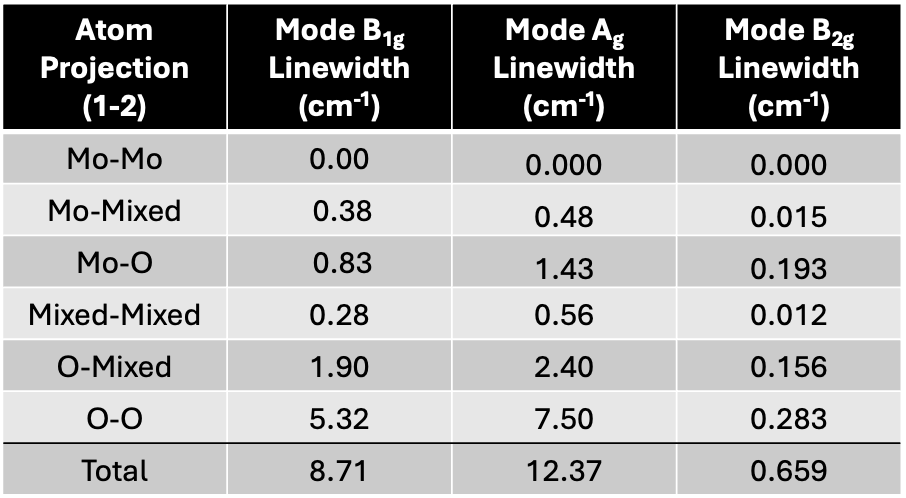

**Figure S2.1:** *Ab initio* mode-resolved three-phonon scattering analysis for Raman-active modes. Decomposition of the anharmonic phonon linewidth Γ for three characteristic Raman-active modes at the Γ-point: mode B_1g_ (ω₀ = 640 cm⁻¹, Γ = 8.7 cm⁻¹), mode A_g_ (ω₀ = 762 cm⁻¹, Γ = 12.4 cm⁻¹), and mode B_2g_ (ω₀ = 1013 cm⁻¹, Γ = 0.66 cm⁻¹), calculated at T = 300 K using phono3py. (a,d,g) Daughter phonon frequency distributions (ω₁ vs ω₂) weighted by their contribution to the total linewidth. The dashed grey lines at 180 cm⁻¹ demarcate the acoustic–optical boundary. The cyan star marks the parent mode frequency. (b,e,h) Momentum-space distribution of scattering processes, showing the linewidth contribution as a function of normalized momentum transfer |**q**|/q_BZ_; Blue and orange curves denote in-plane (q∥) and out-of-plane (q⊥) components, respectively. (c,f,i) Daughter phonon character distributions showing the oxygen projection of daughter modes 1 and 2. Values near 0 correspond to predominantly Mo-centered vibrations, while values near 1 indicate O-dominated motion. The white grid lines at 1/3 and 2/3 delineate Mo-Mo, Mixed, and O-O character zones. Color intensity represents the scattering rate contribution written in linewidth notation (cm⁻¹ ) of the triplets of that character bin.

**Section S3: FTIR measurements of isotopic α-MoO_3_ flakes**


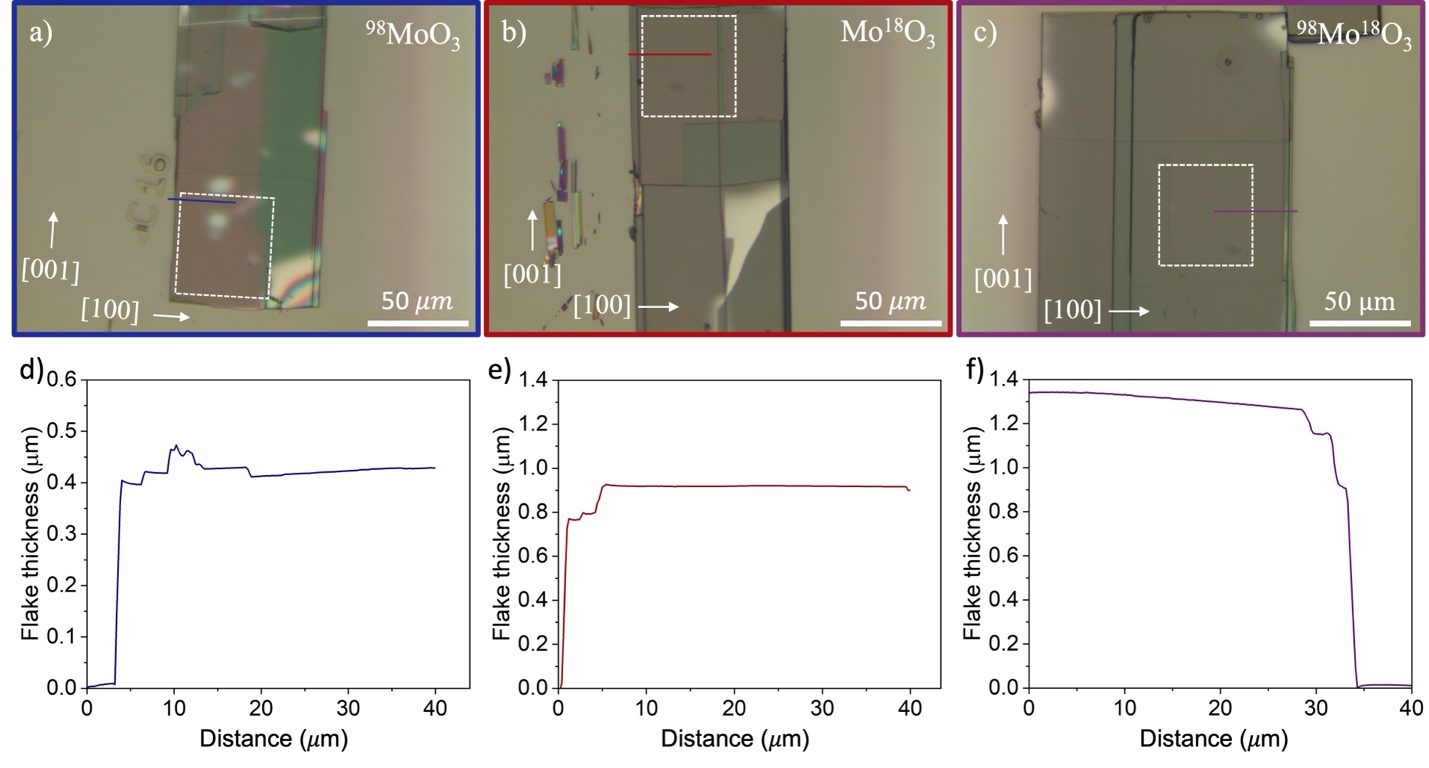


**Figure S3.1:** Visible microscope images of isotopic α-MoO_3_ flakes (a-c) and their respective thicknesses extracted from AFM measurements (d-f). In a-c), the dashed white box insets are where FTIR reflection spectra was collected, and the colored bold lines are where the AFM measurement was taken.

Flakes of each isotopic variation were exfoliated with sufficiency surface area for FTIR measurements (**Figure S3.1**). The polarized FTIR reflection spectra are plotted with frequency markers that approximate the RB_2_ TO phonon frequencies for the respective isotopic enrichments, shown in **Figure S3.2a**. We approximate the onset of the RB_2_ as 828 cm^-1^ for ^98^MoO_3_ and 788 cm^-1^ for Mo^18^O_3_ and ^98^Mo^18^O_3_. While these are crude approximations compared to fitting the reflection spectra with the TO LO model, we identify the expected phonon redshift from ^18^O enrichment of ~ 40 cm^-1^ and validate the similar redshifts between Raman and IR active phonons. Shifting focus to the RB_3_ expanded in **Figure S3.2b**, the redshifts are further validated in the RB_3_ where TO phonon absorption (dotted black line) is present at 914 cm^-1^ for both Mo^18^O_3_ and ^98^Mo^18^O_3_ but much less visible for ^98^MoO_3_ at 966 cm^-1^. While the LO phonon is not IR-active, its spectral position closely correlates with the upper frequency limit of the RB_3_ where the HPhP branches converge. At this convergence, the DOS across wavevectors is significantly higher; resulting in a reflection peak. This correlative identification of the RB_3_ LO phonons (dashed black line) are identified at 1000 cm^-1^ for ^98^MoO_3_ and 954 cm^-1^ for Mo^18^O_3_ and ^98^Mo^18^O_3_. These TO LO phonon frequencies show good agreement with the reported value for naturally abundant α-MoO_3_ at 956.7 cm^-1^ and 1006.9 cm^-1^, respectively^[2]^. Since this correlative LO peak is dependent on the HPhP DOS, we believe the proximity of the crystal edges and fractures serve as a scattering site to excite the HPhPs.

The out-of-plane phonons were excited by collecting reflection spectra at grazing incidence (**Figure S3.2c**), observed in the smaller ratio of reflectance between the RB_2_ and the reflection peak near the LO phonon of the RB_3_. Further investigation of this regime (**Figure S3.2d**) reveals a more accurate identification of the correlative LO reflection peak for ^98^MoO_3_ at 1004 cm^-1^ with respect to the naturally abundant frequency at 1006.9 cm^-1 [2]^. We also attribute the higher reflectivity from the correlative LO peak due to the grazing incidence illumination. At such a shallow angle, total internal reflection is achieved on the surface and creates evanescent fields which sufficient momenta to excite the HPhPs. Due to the nosier spectra from a grazing angle of incidence, the RB_3_ TO phonons are more difficult to discern. Nevertheless, we show excellent agreement between the reported values in literature and the ~ 5 % redshifted phonons. Furthermore, these values are in good agreement with the predicted phonon frequencies reported in Table 1 that were slightly tuned with near-field data.


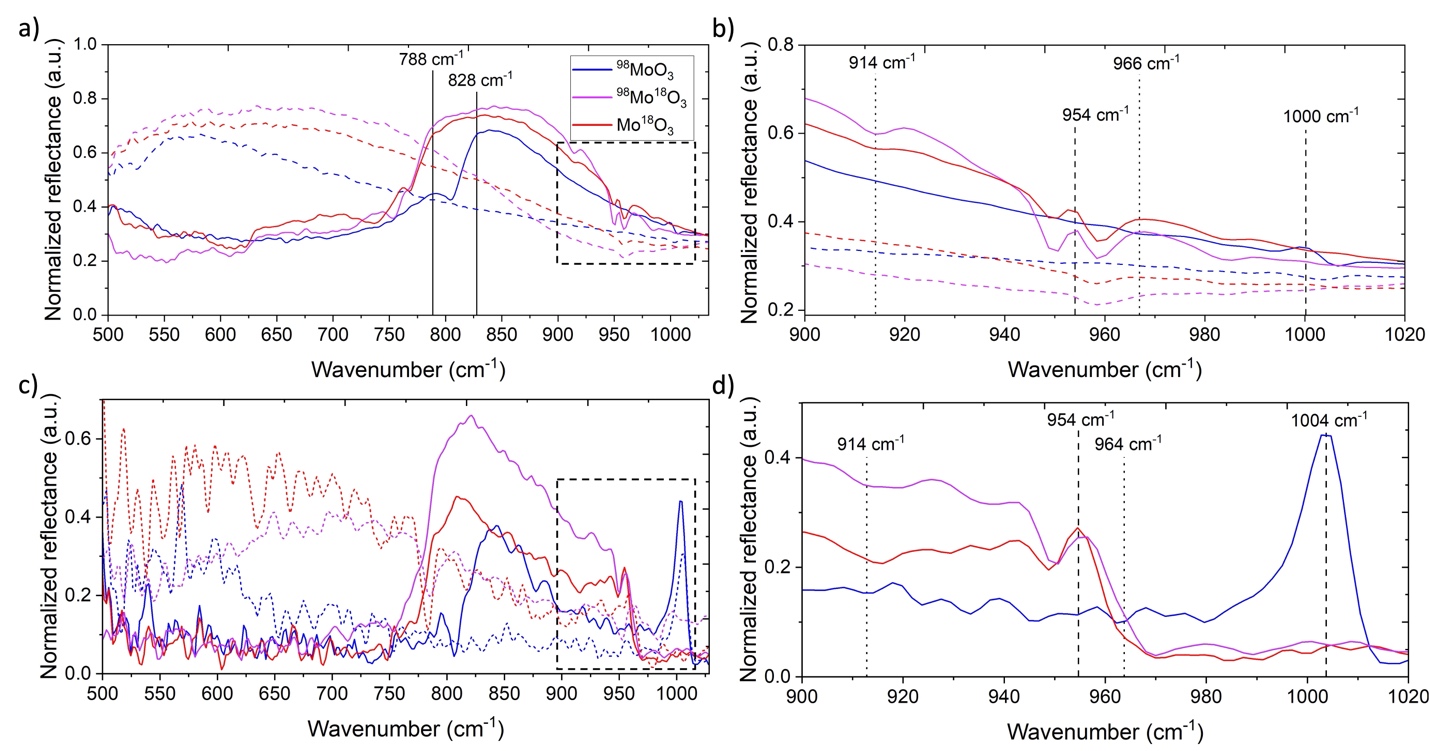


**Figure S3.2:** FTIR reflection spectra taken at near-normal incidence with the 15x Bruker GAO and the RB_2_ TO phonons labeled (a) and an expanded view of phonons in the RB_3_ (b). Reflection spectra taken at grazing incidence with the 15x Bruker GAO (c) and an expanded view of the same phonons in the RB_3_ (d). The vertical lines used in b) and d) correspond to the same phonons for equivalent line styles (i.e. solid, dashed, and dotted). The solid (dashed) line spectra is the linearly polarized reflection taken along the [100] ([001]) crystallographic axis.

**Section S4: *Ab Initio* IR-active phonon modes and linewidths**

In the phonon dispersion of α-MoO₃ (Figure S1.1), the IR-active TO modes that form the three MIR Reststrahlen bands (RB_1-3_) reside in the O-dominated portion of the optical manifold above ≈ 500 cm⁻¹, consistent with the eigenvector projections in Figure S1.2 and the real-space TO eigen-displacements shown in Figure S1.3. For each isotopic sample, we extract the Γ-point TO and LO phonon frequencies and the corresponding TO linewidths from the DFPT calculated within the harmonic and three-phonon scattering formalisms mentioned previously (**Table S4.1-4**). These tables list the *ab initio* TO LO frequencies and linewidths that underlie the dielectric-function discussion and transfer-matrix modeling in the main text. The damping for each of the principal RB’s TO phonon discussed in this work were plotted as a function of isotopic enrichment in **Figure S4.1**. Similar to the Raman linewidths (Figure 1b-d), we predict a systematic reduction in the IR-active TO phonon scattering linewidths, particularly for RB_1_ and RB_2_, due to ^18^O enrichment from first principles calculations, whereas ^98^Mo enrichment produces only minor changes. Precisely, we calculate the percent linewidth reduction between the averages of ^16^O and ^18^O isotopes resulting in 11.5 %, 16.8 %, and 13.2 % for RB_1-3_, respectively. These trends translate into modest lifetime improvements for the mid- and high-frequency RBs that drive the observed increase in HPhP Q-factors for ^18^O-enriched samples in the main text.

**Table S4.1:** *Ab Initio* dielectric function parameters of naturally abundant α-MoO_3_**.**

**Table S4.2:** *Ab Initio* dielectric function parameters of ^98^MoO_3_

**Table S4.3:** *Ab Initio* dielectric function parameters of Mo^18^O_3_

**Table S4.4:** *Ab Initio* dielectric function parameters of ^98^Mo^18^O_3_

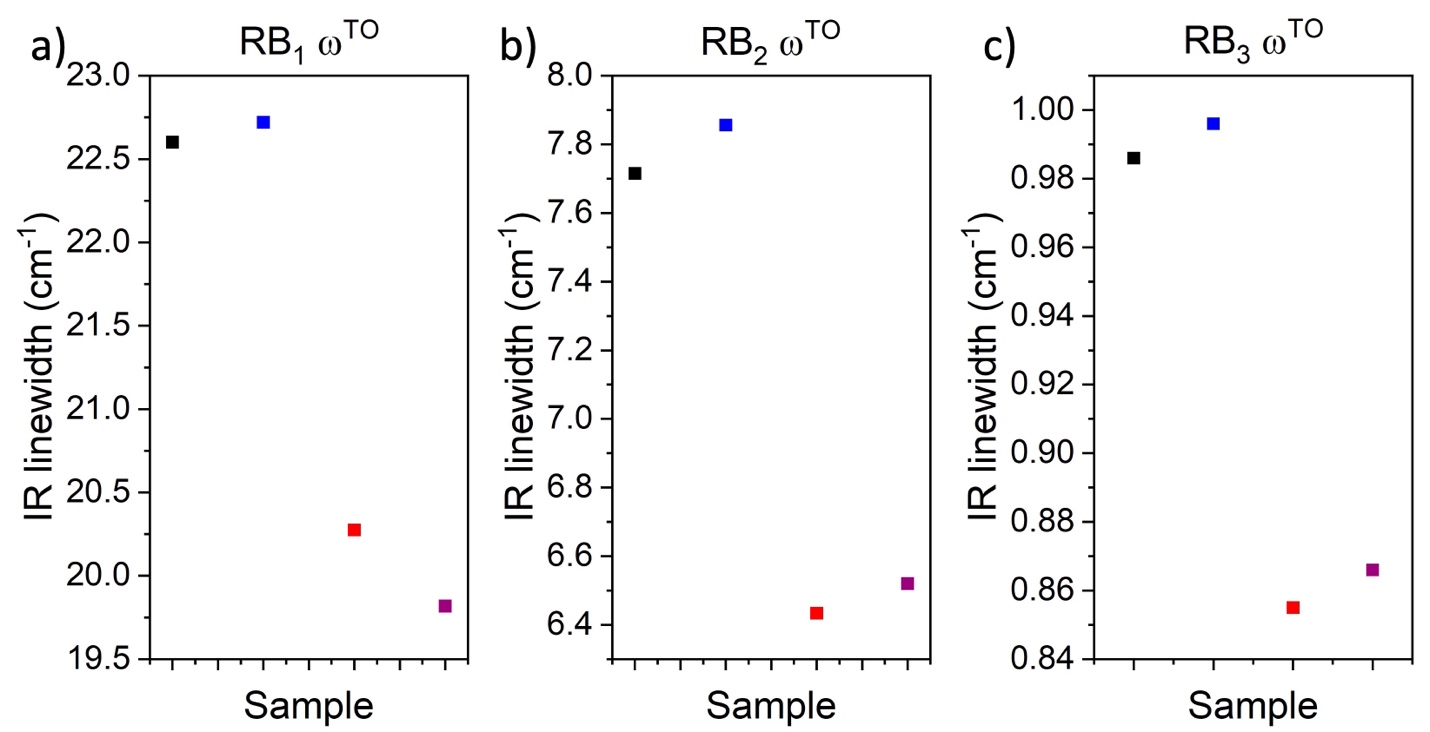


**Figure S4.1:** *Ab Initio* IR-active TO linewidths for each Reststrahlen band across isotopes (a-c).

**Section S5: TDTR measurements**

Section S5.1: Cross-plane thermal conductivity measurements

To measure the cross-plane thermal conductivity of the MoO_3_ flakes we use time-domain thermoreflectance (TDTR). In our TDTR setup, a Ti:sapphire laser with a central wavelength of ∼ 800  nm and a repetition rate of 80 MHz emanates a train of sub-picosecond laser pulses which are split into a pump and a probe path. We modulate the 800  nm pump beam using an electro-optical modulator (EOM) at a frequency of 8.4  MHz. Using a lock-in amplifier and a balanced photodetector, the probe laser detects the reflectivity change due to surface temperature oscillations induced from the modulated pump train and, through use of a mechanical delay stage, measures the temperature decay up to 5.5  ns. These measurements implement a 10 x objective which imposes 1/e^2^ beam diameters of 11.7 µm and 19.3 µm for the probe and pump respectively.

The flakes are coated with an aluminum (Al) transducer in order to convert the optical energy to thermal energy within our sample stack. We use a two layer model to the analytical solution for the radially symmetric heat diffusion model to determine the cross-plane thermal conductivity of MoO_3_ fitting for the thermal boundary conductance between Al and α-MoO_3_ as well as the cross-plane thermal conductivity of α-MoO_3_.^[3,4]^ Our fitting results, shown in **Table S5.1**, indicate a constant thermal conductivity within uncertainty across the different isotopic enrichments. **Figure S5.1** exhibits an example best-fit of the thermal model compared to the ratio of the in-phase and out-of-phase data (–X/Y) for the ^98^MoO_3_ enriched film. The parameters for our thermal model are depicted in **Table S5.2**.

**Table S5.1:** Cross-plane thermal conductivity and thermal boundary conductance fitting results for the α-MoO_3_ films.

|  | $\boldsymbol{\kappa}$ **(W m^-1^ K^-1)^** | **Al-MoO_3_ TBC (MW m^-2^ K^-1^)** | $\mathbf{TBC}$ **lower bound (MW m^-2^ K^-1^)** | $\mathbf{TBC}$ **upper bound (MW m^-2^ K^-1^)** |
| --- | --- | --- | --- | --- |
| Nat-MoO_3_ | \| 2.03 $\pm$.36 \| \| --- \| | \| 58.3 \| \| --- \| | 40.0 | 76.0 |
| Mo^18^O_3_ | 2.08 $\pm$.37 | \| 52.6 \| \| --- \| | 37.5 | 75.4 |
| ^98^MoO_3_ | 2.16 $\pm$.37 | \| 55.3 \| \| --- \| | 40.6 | 81.5 |
| ^98^Mo^18^O_3_ | 2.06 $\pm$.36 | \| 53.9 \| \| --- \| | 37.7 | 76.0 |


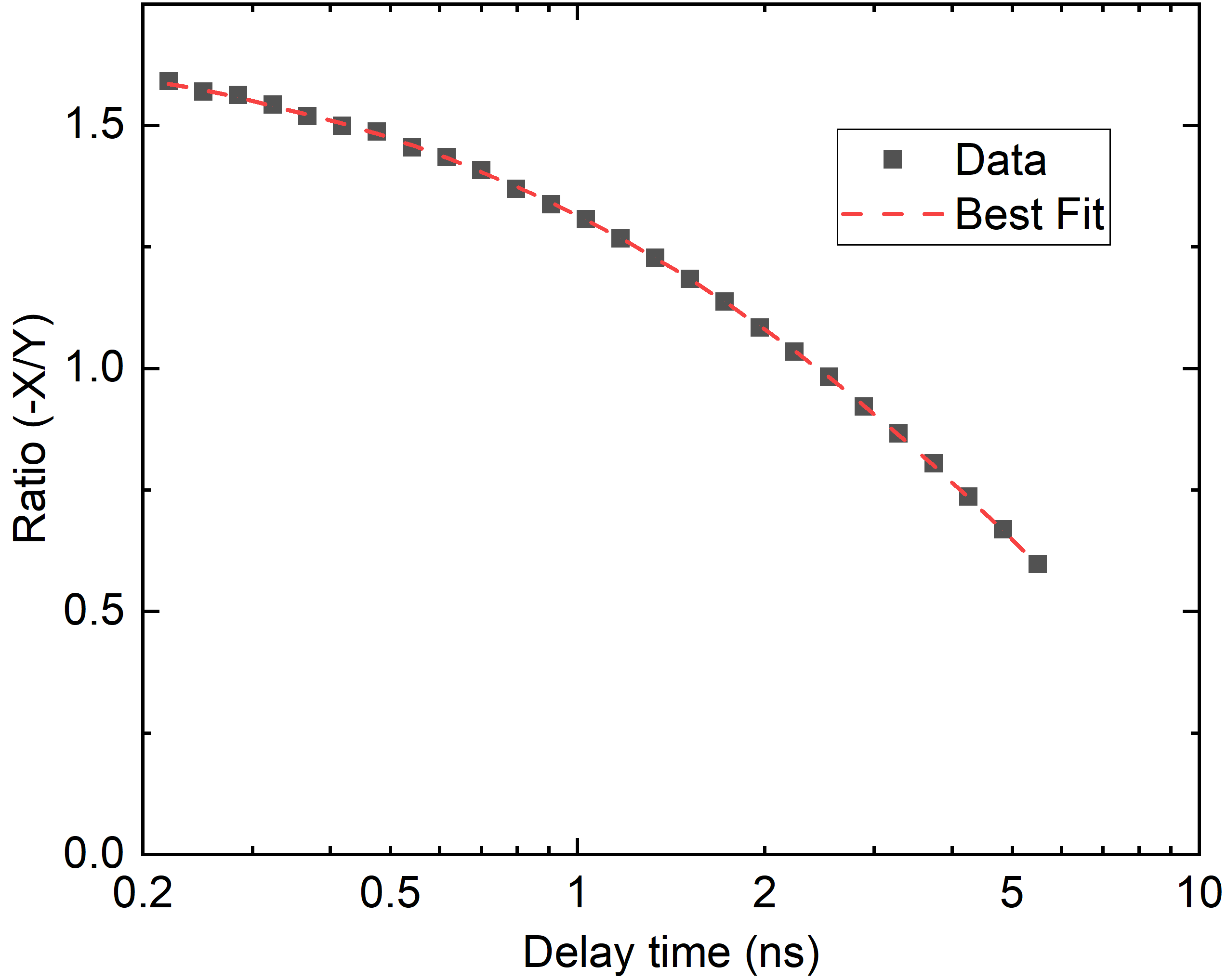


**Figure S5.1:** Example TDTR fit for the ^98^MoO_3_ enriched film.

**Table S5.2:** Parameters used in the thermal model to determine the cross-plane thermal conductivity of α-MoO_3_. The aluminum thermal conductivity and thickness were measured via 4-point probe and picosecond acoustics respectively.^[5]^ We assume the α-MoO_3_ as a semi-infinite substrate due to the large thickness of the flakes ~ 10 µm.

|  | **Thermal conductivity**  **W m^-1^ K^-1^** | **Heat capacity,**  **MJ m^-3^ K^-1^** | **Thickness (nm)** |
| --- | --- | --- | --- |
| Al | 144  (Measured) | 2.43  (Ref ^[S5]^) | 91  (Measured) |
| α-MoO_3_ | Fit | 2.44  (Ref ^[S6]^) | - |

The uncertainty in our TDTR measurements is calculated using **Eq. S5.1** wh­­­­ich accounts for spot-to-spot deviations as well as uncertainty in the fitting assumptions.^[8]^

| Δ = $\sqrt{(\sigma^{2})+(\sum_{i} {}_{i}^{2})+{(\sigma}_{C}^{2})}$ | [Eq.S5.1] |
| --- | --- |
|  |  |

where Δ is the total uncertainty, σ is the standard deviation among multiple measurements across different spots, ${}_{i}$ is the uncertainty due to an individual parameter, and $\sigma_{c}$ is the contour uncertainty due to fitting assumptions^[9,10]^. For our uncertainty we assume a 5 % uncertainty in aluminum heat capacity, aluminum thermal conductivity, and the thickness of the aluminum transducer. This range of uncertainty is typical in most TDTR measurements.^[8]^ We calculate the uncertainty due to our fitting procedure via the method outlined by Feser *et al*.^[11]^ comparing the residual between our fit and the experimental data for the fitted thermal parameters in our model. This allows for a better estimation of the uncertainty in the Al-MoO_3_ TBC, which has much lower sensitivity than the α-MoO_3_ thermal conductivity due to it being a lower resistance. For all of our measurements, our fitting procedure has low residuals around 0.005. Thus, for these measurements, we use a residual threshold of 0.01 added to the best fit residual to bound our fit indicating that any fit with a residual under ~  0.015 is acceptable and contributes to our uncertainty.

Section S5.2: In-plane thermal conductivity measurements

For our in-plane thermal conductivity measurement we use a modified procedure to our cross-plane TDTR measurements to maximize the sensitivity to the in-plane thermal conductivity of α-MoO_3_. First, we utilize a transducer of 15 nm Al/80 nm Ti, where the low thermal conductivity of titanium increases sensitivity to in-plane heat spreading in MoO_3_ while the 15 nm Al surface layer allows us to maintain good thermoreflectance at our probe wavelength^[12]^. This transducer has been utilized in previous works to maximize sensitivity to in-plane thermal conductivity.^[12]^ To further our increase our sensitivity to the in-plane heat spreading we use a 20 x objective (4.4  x  4.4 µm 1/e^2^ diameter spot size) and low modulation frequency measurements at 1.2  MHz. This procedure of small spot size and low modulation frequency measurements to maximize sensitivity to in-plane thermal conductivity has also been described in previous works.^[12,13]^ For these measurements we utilize a three-layer model incorporating both layers of the transducer and the α-MoO_3_. We measure the properties of the transducer by performing 8.4  MHz modulation frequency measurements on two calibration samples with known thermal properties: Al_2_O_3_ and a-SiO_2_, fitting for the thermal conductivity of the aluminum and titanium. For our model we assume an Al/Ti thermal boundary conductance of 3  GW m^-2^ K^-1^ which has been used in previous works.^[12,14–16]^ Next, we perform 8.4  MHz modulation frequency measurements on our α-MoO_3_ samples fitting for the cross plane thermal conductivity of the α-MoO_3_ and the Ti/MoO_3_ thermal boundary conductance. Using these thermal properties, we fit solely for the radial thermal conductivity of the α-MoO_3_ layer. The results with the assumed values for our sample stack depicted are in **Table S5.3**; the results for all of these measurements are given in **Table S5.4**. The uncertainty for each of the fitted values is calculated using **Eq. S5.1** following the procedure described for the cross-plane thermal conductivity uncertainty analysis.

**Table S5.3:** Parameters used in the thermal model to determine the in-plane thermal conductivity of α-MoO_3_. We assume the α-MoO_3_ as a semi-infinite substrate due to the large thickness of the flakes ~ 10 µm.

|  | $\boldsymbol{\kappa}_{\boldsymbol{\parallel}}$  **(W m^-1^ K^-1^)** | $\boldsymbol{\kappa}_{\boldsymbol{\perp}}$  **(W m^-1^ K^-1^)** | **Heat capacity,**  **MJ m^-3^ K^-1^** | **Thickness (nm)** | **Thermal boundary conductance**  **(W m^-2^ K^-1^)** |
| --- | --- | --- | --- | --- | --- |
| Al | Isotropic | 118.9  (Measured) | 2.43  (Ref. ^[6]^) | 15 | - |
| Al/Ti | - | - | - | - | 3e9  (Refs. ^[12,14–16]^) |
| Ti | Isotropic | 14.63  (Measured) | 2.36  (Ref. ^[17]^) | 88 | - |
| Ti/MoO_3_ | - | - | - | - | Fit |
| α-MoO_3_ | Fit | Fit | 2.44  (Ref. ^[7]^) | - | - |

**Table S5.4:** In-plane thermal conductivity results for the α-MoO_3_ films including other thermal parameters that were used in the fitting procedure.

|  | $\boldsymbol{\kappa}_{\boldsymbol{\parallel}}$  **(W m^-1^ K^-1)^** | $\boldsymbol{\kappa}_{\boldsymbol{\perp}}$  **(W m^-1^ K^-1^)** | **Ti-MoO_3_ TBC**  **(MW m^-2^ K^-1^)** | $\mathbf{TBC}$ **lower bound (MW m^-2^ K^-1^)** | $\mathbf{TBC}$ **upper bound (MW m^-2^ K^-1^)** |
| --- | --- | --- | --- | --- | --- |
| Nat-MoO_3_ | \| 9.8 $\pm$2.7 \| \| --- \| | \| 2.22 $\pm$.30 \| \| --- \| | 92.4 | 59.9 | 204.5 |
| Mo^18^O_3_ | 9.9 $\pm$.2.6 | \| 2 $\pm$.29 \| \| --- \| | 81.1 | 47.6 | 161.6 |
| ^98^MoO_3_ | 10.4 $\pm$2.94 | \| 2.29 $\pm$.34 \| \| --- \| | 97.9 | 55.3 | 214.8 |

**Section S6: *Ab Initio* thermal conductivity and isotope mass-variance effects**

The lattice thermal conductivity tensor was evaluated by solving the linearized phonon Boltzmann transport equation (BTE) within the relaxation-time approximation (RTA),

| $\kappa_{\alpha\beta}\left( T \right)= \left( \frac{1}{NV} \right)\sum_{\mathbf{q},j} C_{\mathbf{q}j} v_{\mathbf{q}j,\alpha}\bigotimes v_{\mathbf{q}j,\beta} \tau_{\mathbf{q}j}(T) ,$ | [Eq.S6.1] |
| --- | --- |

where $V$ is the crystal volume and $N$is the number of q-points, and the sum runs over wavevectors **q** and phonon branches s. The mode heat capacity $C_{\mathbf{q}j}$ and group velocity components $v_{\mathbf{q}j,\alpha}$ are computed from the harmonic IFCs, while $\tau_{\mathbf{q}j}(T)$ is the phonon lifetime determined by the total scattering rate. All results in **Figures S6.1-3** were obtained with phono3py using the same second- and third-order interatomic force constants as described in the main Methods, and the linearized BTE was solved at $T=300$ K.

Isotope disorder is treated using the Tamura mass-variance model. For each atomic species $\zeta$, we define the mass-variance parameter

| $g_{\zeta}=\sum_{i} f_{i\zeta}\left( \frac{m_{i\zeta}}{\bar{m_{\zeta}}-1} \right)^{2}$, | [Eq.S6.2] |
| --- | --- |

where $f_{i\zeta}$ and $m_{i\zeta}$ are the fractional abundance and mass of isotope $i$ on sublattice $\zeta$, and $\bar{m_{\zeta}}$ is the isotope-averaged mass. The corresponding isotope-disorder scattering rate $\Gamma_{\mathbf{q}j}^{\mathrm{iso}}$ is added to the intrinsic anharmonic rate $\Gamma_{\mathbf{q}j}^{\mathrm{anh}}$obtained from three-phonon processes, $\Gamma_{\mathbf{q}j}^{\mathrm{tot}}=\Gamma_{\mathbf{q}j}^{\mathrm{anh}}+\Gamma_{\mathbf{q}j}^{\mathrm{iso}}$, with $\tau_{\mathbf{q}j}=1/(2\Gamma_{\mathbf{q}j}^{\mathrm{tot}})$. In all cases, the dynamical matrix is constructed using isotope-averaged atomic masses, while the harmonic and anharmonic IFCs are kept fixed. This procedure captures both the mass-induced softening of phonon frequencies (and associated changes in group velocities) and the additional elastic scattering from mass disorder for each isotope configuration.

**Figure S6.1** summarizes the resulting thermal conductivity tensor components $\kappa_{xx}$, $\kappa_{yy}$, and $\kappa_{zz}$ at 300 K for unenriched, ^98^Mo-enriched, ^18^O-enriched, and doubly enriched samples, with and without isotope scattering. The calculated in-plane thermal conductivity for natural-MoO_3_ $\kappa_{\parallel}\approx10 W m^{-1}K^{-1}$ shows excellent quantitative agreement with our experimental TDTR measurements reported in Section S5 ($9.8\pm2.7 W m^{-1}K^{-1}$). This agreement validates the accuracy of the underlying harmonic and anharmonic force constants used here to decompose the thermal transport properties.

The strong anisotropy $\kappa_{xx}>\kappa_{yy}\gg\kappa_{zz}$ reflects the layered orthorhombic structure and is consistent with the TDTR measurements in the main text. Eliminating the Mo isotope disorder (⁹⁸MoO₃) produces only a modest increase in κ, whereas ¹⁸O enrichment lowers κ in all directions despite the removal of O-isotope scattering, demonstrating that the dominant effect of ¹⁸O is the ≈ 12.5 % oxygen mass increase and corresponding reduction in phonon group velocities rather than additional elastic disorder.

**Figure S6.1:** *Ab initio* thermal conductivity values in isotopically enriched along each principal crystallographic direction α-MoO_3_. Calculated lattice thermal conductivity tensor components κ_xx_, κ_yy_, and κ_zz_ at 300 K for unenriched (natural isotope abundance, black), ⁹⁸Mo-enriched (⁹⁸MoO₃, blue), ¹⁸O-enriched (Mo¹⁸O₃, red), and doubly enriched (⁹⁸Mo¹⁸O₃, purple) samples. Open squares denote calculations including only three-phonon anharmonic scattering, while filled squares include both anharmonic and isotope scattering contributions.

The microscopic origin of this behavior is clarified in **Figure S6.2**, which decomposes κ into frequency-, momentum-, and species-resolved contributions, aided by the atom-projected phonon dispersion in Figure S1.2. At 300 K, acoustic modes (ω < 180 cm⁻¹) carry the majority of the heat current in all directions—about 68 % of κₓₓ, 60 % of κᵧᵧ, 70 % of κzz, and 65 % of κ∥ = (κₓₓ+κᵧᵧ)/2—while optical modes contribute the remaining 30–40 %. The in-plane conductivity is dominated by long-wavelength acoustic modes with mixed Mo–O character at small $\mid\mathbf{q}\mid/\mathbf{q}_{\mathrm{BZ}}$, whereas κ_zz_, though still primarily acoustic, receives a more broadly distributed contribution across momentum space, reflecting the weaker interlayer van der Waals bonding along z. The species-projection analysis further shows that modes with substantial participation from both Mo and O, rather than purely Mo- or purely O-like vibrations, are the main heat carriers in both κ∥ and κ_zz_, which explains their sensitivity to changes in the oxygen mass under ¹⁸O enrichment.

**Figure S6.2:** Mode-resolved decomposition of the calculated lattice thermal conductivity in α-MoO₃ at 300 K. (a, d) Frequency-resolved κ contributions showing acoustic (blue, ω < 180 cm⁻¹) and optical (orange, ω > 180 cm⁻¹) phonon contributions for in-plane κ_∥_ = (κ_xx_ + κ_yy_)/2 (a) and cross-plane κ_zz_ (d). The acoustic/optical cutoff is marked by the dashed vertical line, consistent with the acoustic and optical bands in Figure S1.1. (b, e) Momentum-resolved κ contributions as a function of normalized wavevector magnitude |q|/q_BZ_, decomposed by q-vector orientation into in-plane (q_∥_, blue) and out-of-plane (q_z_, orange) components for κ_∥_ (b) and κ_zz_ (e). (c, f) Species-resolved κ contributions as a function of oxygen character P_O_, where P_O_ is the squared eigenvector projection onto oxygen atoms summed over all O sites. Vertical dashed lines at P_O_ = 1/3 and 2/3 delineate Mo-dominated (P_O_ < 1/3), mixed (1/3 ≤ P_O_ ≤ 2/3), and O-dominated (P_O_ > 2/3) character regions for κ_∥_ (c) and κ_zz_ (f). The analysis reveals that in-plane thermal transport is dominated by long-wavelength (small |q|) acoustic modes with mixed Mo–O character, while cross-plane transport shows a more distributed contribution across momentum space, reflecting the weak interlayer van der Waals bonding along the z-direction.

Finally, **Figure S6.3** recasts the κ changes under isotope enrichment in terms of their basic BTE ingredients via a component-substitution analysis. By recomputing κ while substituting, one at a time, the mode heat capacities, squared group velocities, anharmonic scattering rates, or isotope-scattering rates from the enriched cases into the unenriched reference (with $\Gamma^{\mathrm{iso}}=0$), we isolate their individual contributions to $\Delta\kappa$. The results show that ¹⁸O enrichment affects κ primarily through reduced group velocities, with only minor changes arising from heat capacity and intrinsic anharmonic scattering, while the explicit isotope-disorder term mainly reflects the removal of Mo mass variance in the ⁹⁸Mo-enriched case and the predicted percent enrichment of ^18^O. Further investigation of the thermal conductivity along the [100] and [001] could demonstrate the preferential direction of in-plane heat transport and isotopic enrichment could further enhance that degree of anisotropy. The deterministic design of a twisted bilayer isotopic α-MoO_3_ heterostructure could provide a platform of steerable and directional in-plane dissipation of heat. Figures S6.1-3 represent different reorganizations of the same underlying ab initio BTE dataset.


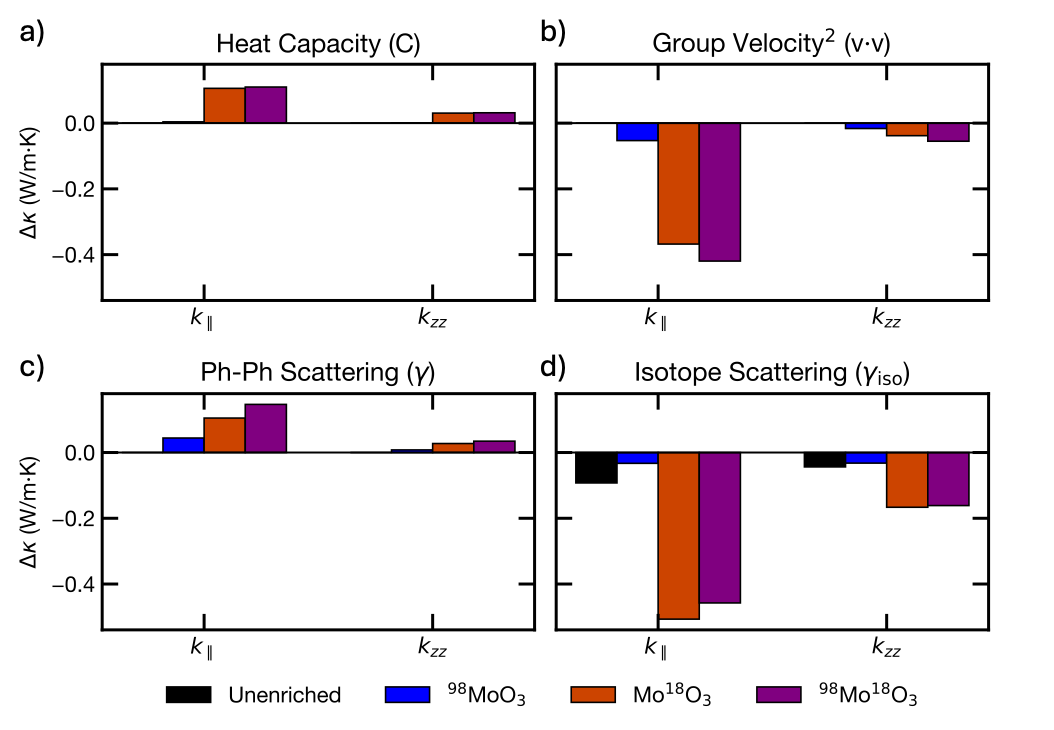


**Figure S6.3:** Decomposition of the calculated changes in thermal conductivity from isotopic enrichment into components of a) phonon heat capacity, b) group velocity, c) scattering rates, and d) isotopic disorder scattering rates. Component-substitution analysis —isolating individual contributions to the thermal conductivity — change κ, Δκ, upon isotope enrichment. Each panel shows the change in in-plane (κ_∥_ = (κ_xx_ + κ_yy_)/2) and cross-plane (κ_zz_) thermal conductivity when only the specified component is substituted from the enriched case, with all other components held at their unenriched baseline values (with γ_iso_ = 0). Colors denote unenriched (black), ⁹⁸Mo-enriched (blue), ¹⁸O-enriched (red), and doubly enriched (purple) configurations.

**Section S7: s-SNOM sample images and AFM measurements**


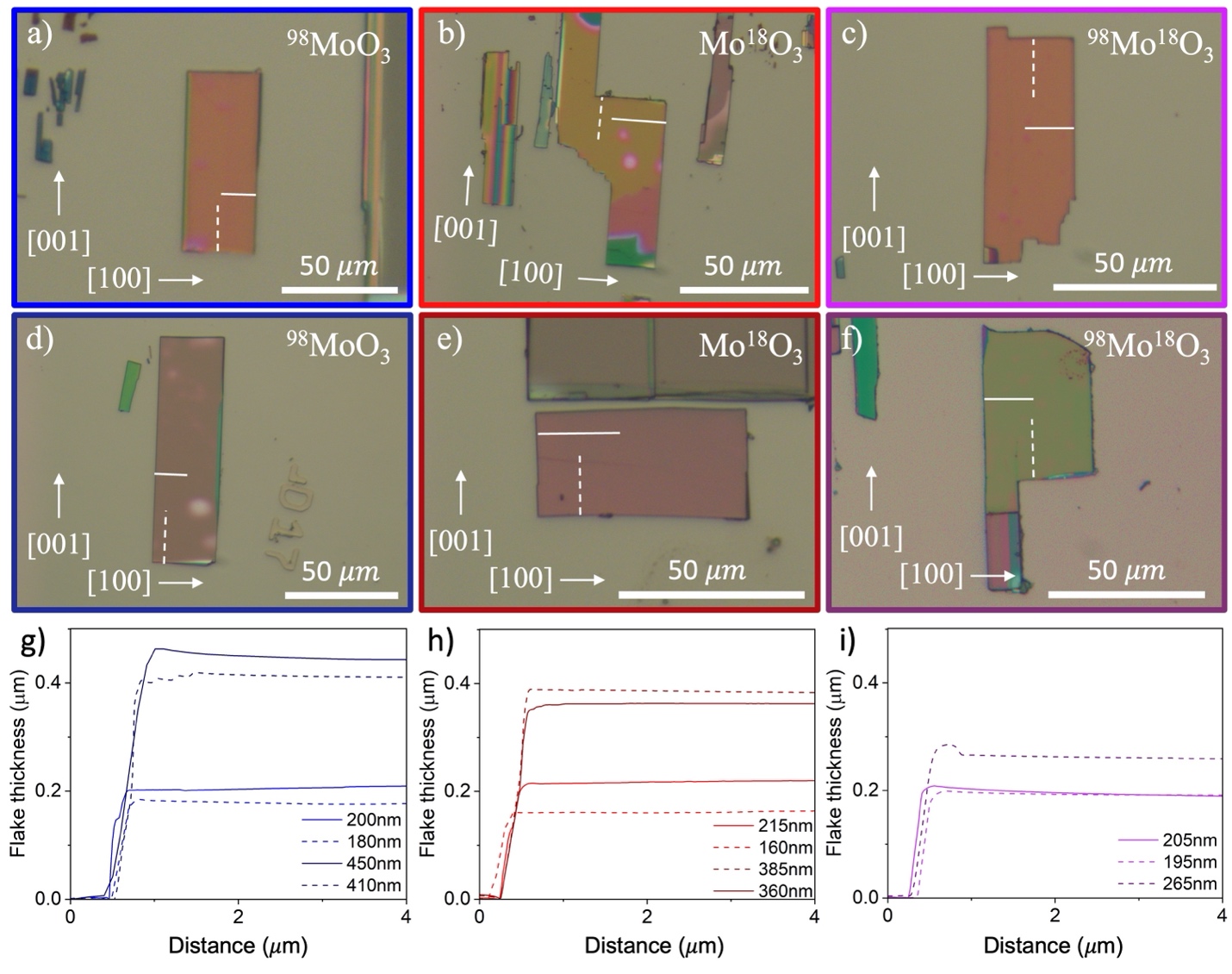


**Figure S7.1:** Visible light microscope images of the thinner (a-c) and thicker (d-f) set of isotopic α-MoO_3_ flakes prepared for s-SNOM measurements with their respective thicknesses mapped from AFM and plotted in (g-i). The solid (dashed) line are line scans taken along the [100] ([001]) crystallographic direction. The darker color scheme (thicker flakes) corresponds to the samples in (d-f).

**Section S8: FFT analysis of HPhPs in real and momentum space**

We extract the frequency dependent line profiles of HPhPs propagating in each isotopic slab of α-MoO_3_ from the s-SNOM images. The beginning of the line scan is taken from the flake edge by mapping the AFM s-SNOM data and coinciding the same pixels in the near-field data to ensure that the profiles as a function of distance from the flake edge. In this section, we examine the line profile extracted from an HPhP excited at 930 cm^-1^ propagating along the [001] in the RB_3_ of a ~ 400 nm Mo^18^O_3_ flake. First and foremost, the raw HPhP profile is put through a high-frequency pass FFT filter to exclude any low frequencies present from the far-field excitation source (a bandpass FFT filter is optional to exclude very high frequencies from a low signal-to-noise ratio) and the DC offset is removed from the FFT. The resulting HPhP profile is seen in **Figure S8.1a**, where the line scan is symmetric about 0 amplitude, the far-field oscillations have vanished, and the near-field profile is still preserved. Aside from the additional oscillations in the first fringe, it is clear that both edge-launched ($\lambda_{P}$) and tip-launched ($\frac{\lambda_{P}}{2}$) HPhPs are present. Following the far-field correction, we scale the near-field amplitude by multiplying a geometric correction factor ($\sqrt{x}$) to account for the radial propagation of a point-launched HPhP. With the HPhP fully prepped for analysis, we apply an FFT with a Hanning window and minimal zero-padding to make the FFT peaks as close to symmetric as possible. The resulting Fourier spectra, F(x), in **Figure S8.1b** displays three distinct peaks to which we fit the following Lorentzian profile to each peak

| F(k) = F_0_ + $\frac{2A}{\pi}(\frac{w}{4(k-k_{c})^{2}}+w^{2})$ | [Eq.S8.1] |
| --- | --- |
|  |  |

where k is the wavevector, k_c_ is the center wavevector of the fitted peak, $w$ is the full-width half maximum (FWHM) of the fitted peak, A is the area of the peak, and F_0_ is the vertical offset. For each fitted peak, the complex wavevector of each modal order of HPhPs present can be extracted by $Re\left( k \right)=k_{c}$ and $Im\left( k \right)=FWHM$. Given by the relationship from edge or tip-launched HPhPs, the $Re\left( k \right)$ of a tip-launched HPhP is twice the frequency of the edge-launched counterpart. Such a frequency relationship is clear in **Figure S8.1b**, where the edge and tip-launched peaks are clearly identified with an additional higher frequency peak present. The free-space normalization of the first and third peaks are included in the data set plotted in **Figure 3e** at 930 cm^-1^, confirming their identification as the *l* = 0 and 1 edge-launched HPhPs. At this point, we calculate the Q-factors from the complex from the complex HPhP wavevector extracted from the Fourier spectra.

To ensure that Q-factors extracted from the Fourier spectra are accurate, we investigate the real and imaginary components independently and compare them with the real-space extracted values. The real-space counterparts are calculated from the Fourier spectra (inset values in **Figure S8.1b**) to the polariton wavelength and propagation length by $\lambda_{P}=\frac{1}{Re\left( k \right)}$ and $L_{P}=\frac{1}{Im\left( k \right)}$, respectively. To compare these values to the direct extraction of the HPhP wavelength and propagation length, we take the scaled HPhP line profile prior to applying the FFT and instead, perform a FFT bandpass filter. Here, we selectively filter the line profile for either the *l* = 0 or 1 order and plot them in **Figure S8.1c** and **Figure S8.1d**, respectively. For the *l* = 0 mode, we filter both the edge and tip-launched frequencies together since their independent filtering led to an inconsistent frequency with respect to the raw line profile. Both FFT filtered HPhP profiles were fitted the damped sine function

| S($\omega$) $\sigma_{3}$ = S_0_ + $A\frac{e^{-\frac{2x}{L_{P}}}}{\sqrt{x}}\sin\left( \frac{4\pi\left( x-x_{a} \right)}{\lambda_{P}} \right)+B\frac{e^{-\frac{x}{L_{P}}}}{x}\sin\left( \frac{2\pi\left( x-x_{b} \right)}{\lambda_{P}} \right)$ | [Eq.S8.2] |
| --- | --- |
|  |  |

where A (B) is the amplitude of the tip (edge) -launched HPhP, $x_{a(b)}$ is the phase shift applied to the tip (edge) -launched HPhP, and S_0_ is the vertical offset. For the *l* = 0 mode, both A and B are non-zero, but we fix A=0 for the *l* = 1 mode since we previously validated its wavevector as an edge-launched mode with the HPhP dispersion. We observe excellent agreement between both the direct extraction of the *l* = 0 mode wavelength and propagation length and the values calculated from the Fourier spectra. Thus, we employ the Fourier analysis of the HPhP wavevectors due to the presence of multiple higher-order modes in our s-SNOM measurements. We acknowledge the discrepancy in the fit in **Figure S8.1c** which shows a degree of over damping for the edge launched but an under damping for the tip-launched HPhP. This is possibly due to the correction factor we apply to the data before fitting. We provide the real-space evaluation of the *l* = 1 mode here to demonstrate the challenges with reporting propagation lengths or Q-factors of higher-order modes. Their damping is likely underestimated due to the dominance of the fundamental mode upon the fringes that we can resolve from the higher-order modes. Therefore, we can only accurately report the R$e\left( k \right)$ of the higher-order modes.


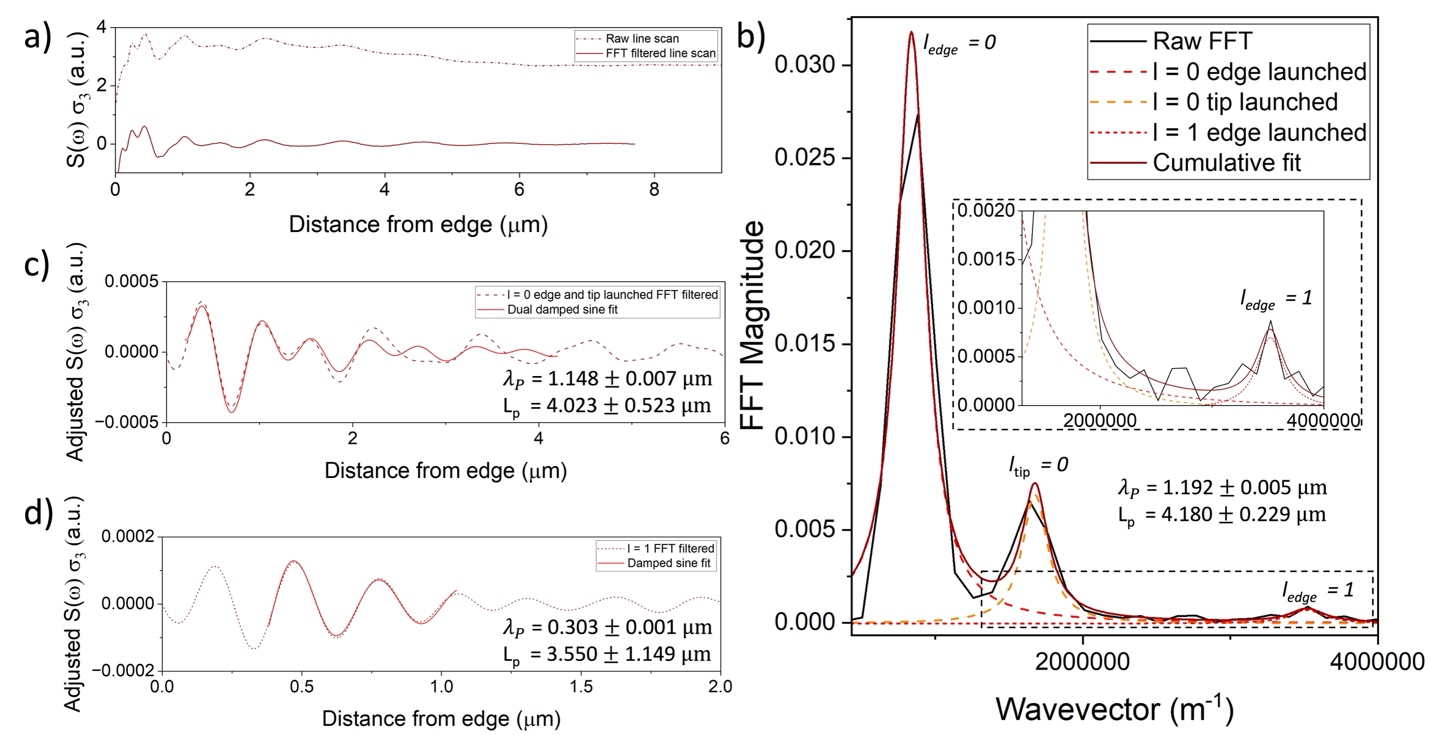


**Figure S8.1:** FFT analysis to extract complex HPhP wavevector from real space and momentum space.

**Section S9: Additional dispersion mapping for various thicknesses**

Here we provide the remaining experimentally mapped dispersion points for the isotopically enriched α-MoO_3_ that was not provided in the main text. As seen with Figure 3, we observe excellent agreement between the TMM calculations and the HPhP wavevector extracted from s-SNOM measurements.


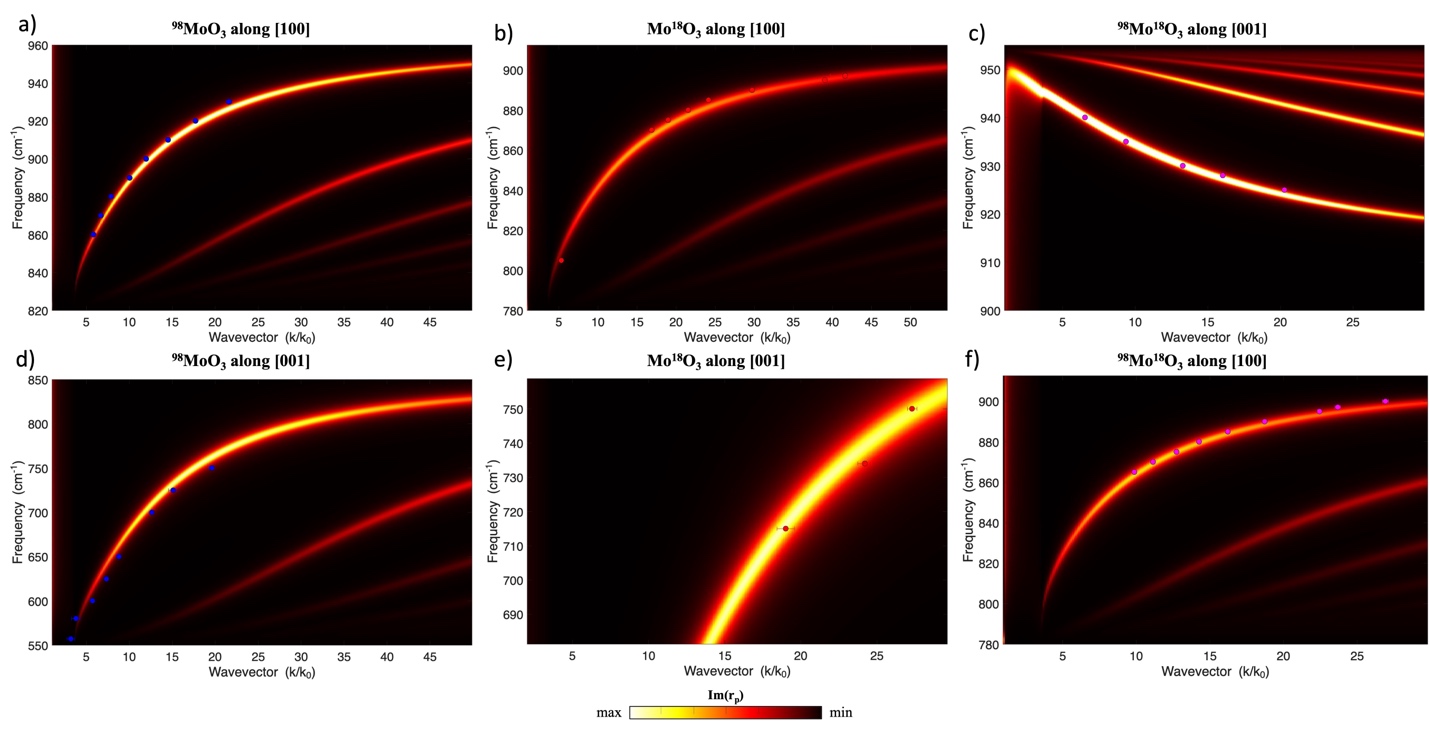


**Figure S9.1:** Experimental dispersion points mapped on TMM calculations of p-polarized Im(r_p_) in a) ~200nm ^98^MoO_3_ along the [100], b) ~200nm Mo^18^O_3_ along the [100], c) ~300nm ^98^Mo^18^O_3_ along the [001], d) ~200nm ^98^MoO_3_ along the [001], e) ~200nm Mo^18^O_3_ along the [001], and f) ~300nm ^98^Mo^18^O_3_ along the [100].

**Section S10. Comparison in dielectric function with literature**

Prior characterizations of ^92^MoO_3_ and ^100^MoO_3_ has been performed by *Schultz et al.*^[18]^ and *Zhao et al.*^[19]^, with their findings provided in **Table S10.1**. For reference to naturally abundant α-MoO_3_, we also include the parameters reported by *Álvarez-Pérez et al*.^[2]^ The high-frequency dielectric constants for the isotopes in this work were manually tuned to fit the higher-order HPhP branches with our experimental data on the *l=2* and *l=3* branches. The resulting summary provides the full spectral tunability available in α-MoO_3_ through selective isotopic enrichment.

**Table S10.1: Summary of dielectric function tunability in isotopically enriched α-MoO_3_**

**Section S11: Additional HPhP Q-factor plots**

The momenta of HPhPs supported in thicker flakes of isotopically enriched α-MoO_3_ were also fully analyzed. As discussed in the main text, the experimental challenges present to accurately compare spectrally shifted HPhPs in the RB_1_ due to isotopic enrichment are present in this set of data (**Figure S11.1a**). With further inspection between **Figure 4a** and **Figure S11.1a**, the Q-factors between ^98^MoO_3_ in the main text and Mo^18^O_3_ are nearly identical in the low wavevector regime. Despite these two samples being significantly distinct in thickness, they were taken during the same day and suffer the same degree of absorption loss from the RB_1_ TO phonon. Therefore, we draw the connection that the RB_1_ TO phonon experiences minimal lifetime improvements from ^18^O enrichment and is in agreement with the prediction of the degree is restriction upon the O(1) atom discussed in Section S1. Within the RB_2_, we observe offset maxima for each isotopic sample which is likely due to the larger discrepancy of sample thickness where the normalized wavevector, k*d, only holds for comparison in small discrepancies of sample thickness^[18]^ (**Figure S11.1b**). However, we still see the improvements in Q-factor in the high-wavevector regime dominated by the expected lifetime improvements due to ^18^O enrichment. As seen in both the RB_2_ and RB_3_, the Q-factors decrease for thicker samples which can be understood through additional scattering losses present as the thickness of the flake is increased (**Figure S11.1c**).

**
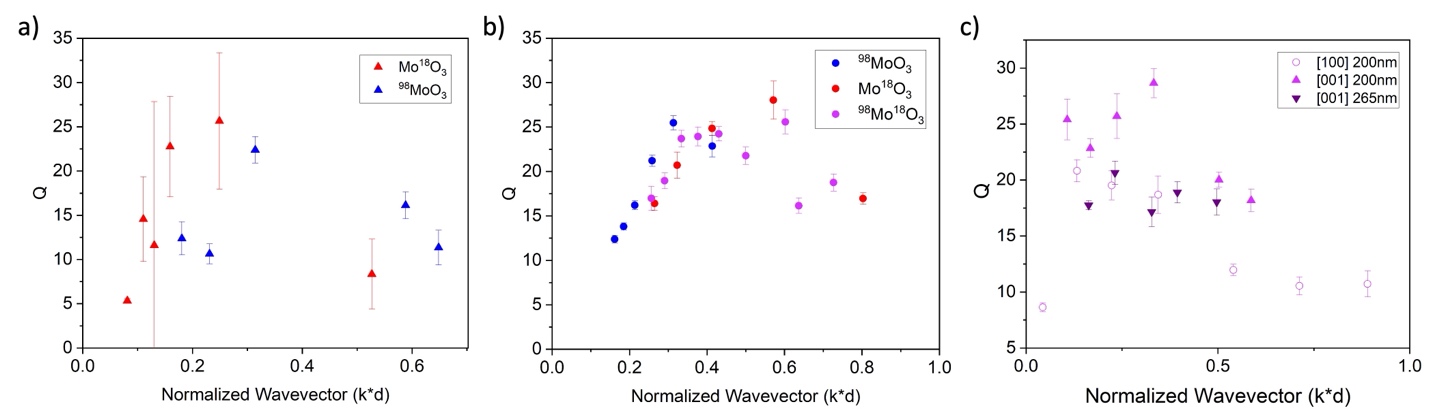
**

**Figure S11.1:** HPhP Q-factors of thicker isotopically enriched α-MoO_3_ flakes in the RB_1_, RB_2_, and RB_3_ for a-c), respectively. Note that the only data set which includes any Q-factors from the thinner flakes is in c).

Here we provide a brief reporting of HPhP Q-factors propagating along the [001] within the RB_1_ of a 200 nm naturally abundant α-MoO_3_ flake shown in **Figure S11.2**. Unlike the RB_2_ and RB_3_, which naturally abundant α-MoO_3_ Q-factors has been previously reported, the RB_1_ has failed to be investigated. Thus, we report these values as a comparison to our isotopic Q-factors. As a reminder, ^98^MoO_3_ is selected in this study to serve a close representation of the mass of naturally abundant α-MoO_3_ without the ^X^Mo isotopic disorder.


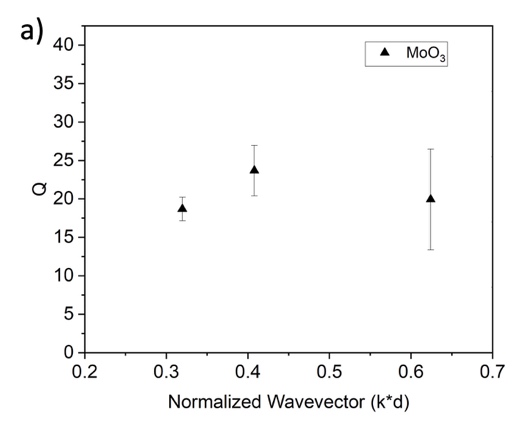


**Figure S11.2:** RB_1_ HPhP Q-factors extracted from a 200 nm naturally abundant α-MoO_3_ flake.

**Section S12: Free space confinement from ^18^O enrichment**

Aside from the Q-factor enhancements originating from the imaginary HPhP wavevector, we also investigate the changes in the real-component HPhP wavevector. Due to the thickness dependence of the complex wavevector shown in Equation 3 of the main text and the invalidity for large thickness discrepancies discussed in Section S11, we restrict this investigation to the data set of samples with comparable thicknesses in which we observe a Q-factor enhancement. Within the RB_2_ of our thinner set of isotopic flakes, we first plot the free space normalized wavevector as a function of the wavenumber differential from the TO phonon defined as

| $\Delta\omega=\omega-\omega^{TO}$ | [Eq.S12.1] |
| --- | --- |
|  |  |

where ω is the excitation wavenumber and ω^TO^ is the TO phonon wavenumber. There is a clear difference between ^16^O and ^18^O flakes in terms of how much their HPhPs are compressing the free-space wavevector as seen in **Figure S12.1a**. While this relationship accounts for the redshift in the TO phonon frequency from ^18^O enrichment, the slight discrepancy in thickness makes for an unjust comparison. Furthermore, for an equal redshift in phonon frequencies between ^98^Mo^18^O_3_ and Mo^18^O_3_, we expect an equal level of confinement but do not observe as such. Since the only distinction between these two isotopes are additional scattering losses which alter the imaginary wavevector component, we conclude these slight differences in confinement are a result of the slight thickness discrepancies skewing this inverted dispersion relationship. This further supports the reasoning discussed in the main text as to why the Q-factors could not be compared in any form of frequency. Therefore, we plot the free-space confinement factor with respect to the thickness normalized wavevector (**Figure S12.1b**). While this is a straightforward representation of free-space confinement, we eliminate any concerns from the dispersion dependence upon thickness. As a result, we still observe a higher degree of confinement in the ^18^O flakes over the ^16^O case (**Figure S12.1c**). Importantly, the increase in confinement is equal across both ^18^O flakes, confirming the expectations described earlier. The implications of this increase in confinement is best understood when considering wavelengths, which are inversely proportional to wavevector. Thus, the HPhPs in the ^18^O flakes achieve higher compression because they achieve the same polariton wavelength for a larger excitation wavelength due to the red shift from ^18^O enrichment.


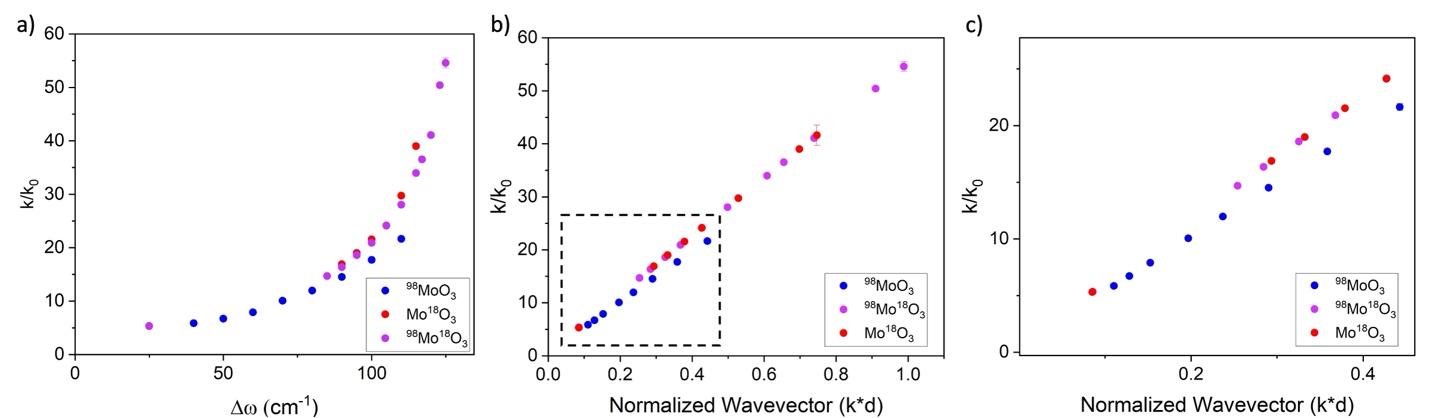


**Figure S12.1:** HPhP confinement of free-space light in the RB_2_ as a function of the difference in wavenumber from the TO phonon (a) and thickness normalized wavevector (b and c); where c) is zoomed into the region comparing all three isotopes.

**S13. Individual RB_3_ Q-factors in isotopically enriched α-MoO_3_ flakes**

**
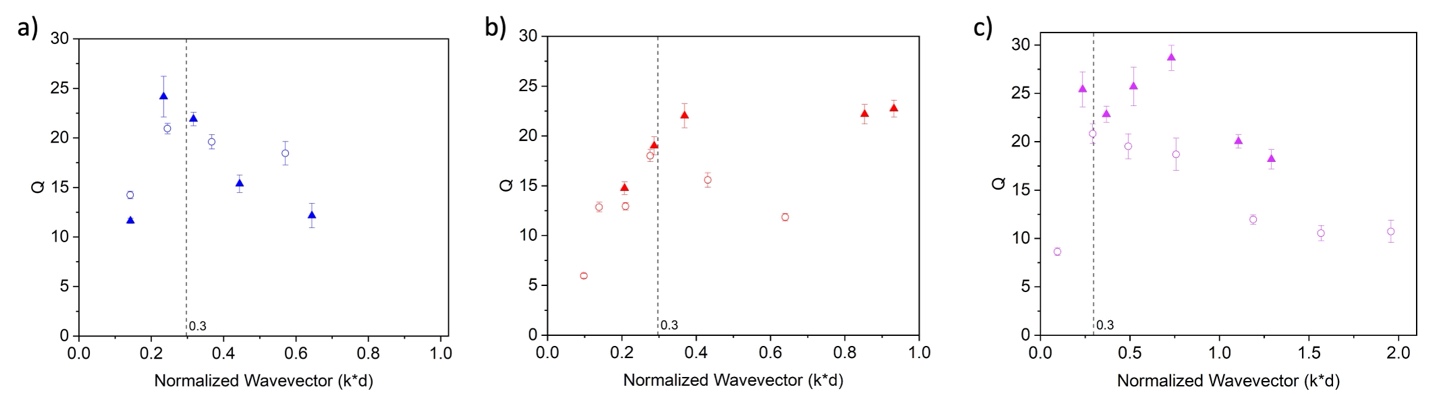
**

**Figure S13.1:** Isotopic HPhP Q-factors within the RB_3_ shown in Fig.4c of the main text plotted in separately for clarity; where a) is ^98^MoO_3_, b) is Mo^18^O_3_, and c) is ^98^Mo^18^O_3_.

**References**

[1] A. Togo, L. Chaput, I. Tanaka, *Phys. Rev. B* **2015**, *91*, 094306.

[2] G. Álvarez-Pérez, T. G. Folland, I. Errea, J. Taboada-Gutiérrez, J. Duan, J. Martín-Sánchez, A. I. F. Tresguerres-Mata, J. R. Matson, A. Bylinkin, M. He, W. Ma, Q. Bao, J. I. Martín, J. D. Caldwell, A. Y. Nikitin, P. Alonso-González, *Adv. Mater.* **2020**, *32*, 1908176.

[3] D. G. Cahill, *Rev. Sci. Instrum.* **2004**, *75*, 5119.

[4] P. E. Hopkins, J. R. Serrano, L. M. Phinney, S. P. Kearney, T. W. Grasser, C. T. Harris, *J. Heat Transf.* **2010**, *132*, DOI 10.1115/1.4000993.

[5] E. A. Scott, S. W. Smith, M. D. Henry, C. M. Rost, A. Giri, J. T. Gaskins, S. S. Fields, S. T. Jaszewski, J. F. Ihlefeld, P. E. Hopkins, *Appl. Phys. Lett.* **2018**, *113*, 192901.

[6] E. H. Buyco, F. E. Davis, *J. Chem. Eng. Data* **1970**, *15*, 518.

[7] M. W. Chase, *J. Phys. Chem. Ref. Data, Monograph 9* **1998**, 1.

[8] M. S. B. Hoque, Y. R. Koh, J. L. Braun, A. Mamun, Z. Liu, K. Huynh, M. E. Liao, K. Hussain, Z. Cheng, E. R. Hoglund, D. H. Olson, J. A. Tomko, K. Aryana, R. Galib, J. T. Gaskins, M. M. M. Elahi, Z. C. Leseman, J. M. Howe, T. Luo, S. Graham, M. S. Goorsky, A. Khan, P. E. Hopkins, *ACS Nano* **2021**, *15*, 9588.

[9] A. Giri, J.-P. Niemelä, C. J. Szwejkowski, M. Karppinen, P. E. Hopkins, *Phys. Rev. B* **2016**, *93*, 024201.

[10] T. W. Pfeifer, H. B. Schonfeld, E. A. Scott, H. T. Aller, J. T. Gaskins, D. H. Olson, J. L. Braun, S. Graham, P. E. Hopkins, *Annu. Rev. Mater. Res.* **2025**, *55*, 37.

[11] J. P. Feser, D. G. Cahill, *Rev. Sci. Instrum.* **2012**, *83*, 104901.

[12] M. R. Islam, P. Karna, J. A. Tomko, E. R. Hoglund, D. M. Hirt, M. S. B. Hoque, S. Zare, K. Aryana, T. W. Pfeifer, C. Jezewski, A. Giri, C. D. Landon, S. W. King, P. E. Hopkins, *Nat. Commun.* **2024**, *15*, 9167.

[13] D. Hirt, Md. R. Islam, Md. S. B. Hoque, W. Hutchins, S. Makarem, M. K. Lenox, W. T. Riffe, J. F. Ihlefeld, E. A. Scott, G. Esteves, P. E. Hopkins, *Appl. Phys. Lett.* **2024**, *124*, 202202.

[14] B. C. Gundrum, *Phys. Rev. B* **2005**, *72*, DOI 10.1103/PhysRevB.72.245426.

[15] R. B. Wilson, D. G. Cahill, *Phys. Rev. Lett.* **2012**, *108*, DOI 10.1103/PhysRevLett.108.255901.

[16] R. Cheaito, K. Hattar, J. T. Gaskins, A. K. Yadav, J. C. Duda, T. E. Beechem, J. F. Ihlefeld, E. S. Piekos, J. K. Baldwin, A. Misra, P. E. Hopkins, A. Sandia National Laboratories (SNL-NM), *Appl. Phys. Lett.* **2015**, *106*, 93114.

[17] D. H. Olson, M. G. Sales, J. A. Tomko, T.-F. Lu, O. V. Prezhdo, S. J. McDonnell, P. E. Hopkins, *Appl. Phys. Lett.* **2021**, *118*, 163503.

[18] J. F. Schultz, S. Krylyuk, J. J. Schwartz, A. V. Davydov, A. Centrone, *Nanophotonics* **2024**, *13*, 1581.

[19] Y. Zhao, J. Chen, M. Xue, R. Chen, S. Jia, J. Chen, L. Bao, H.-J. Gao, J. Chen, *Nano Lett.* **2022**, *22*, 10208.
